# Validation of a LC-MS/MS Method for Quantifying Urinary Nicotine, Six Nicotine Metabolites and the Minor Tobacco Alkaloids—Anatabine and Anabasine—in Smokers' Urine

**DOI:** 10.1371/journal.pone.0101816

**Published:** 2014-07-11

**Authors:** James E. McGuffey, Binnian Wei, John T. Bernert, John C. Morrow, Baoyun Xia, Lanqing Wang, Benjamin C. Blount

**Affiliations:** Tobacco and Volatiles Branch, Division of Laboratory Sciences, National Center for Environmental Health, Centers for Disease Control and Prevention, Atlanta, Georgia, United States of America; The Scripps Research Institute, United States of America

## Abstract

Tobacco use is a major contributor to premature morbidity and mortality. The measurement of nicotine and its metabolites in urine is a valuable tool for evaluating nicotine exposure and for nicotine metabolic profiling—i.e., metabolite ratios. In addition, the minor tobacco alkaloids—anabasine and anatabine—can be useful for monitoring compliance in smoking cessation programs that use nicotine replacement therapy. Because of an increasing demand for the measurement of urinary nicotine metabolites, we developed a rapid, low-cost method that uses isotope dilution liquid chromatography-tandem mass spectrometry (LC-MS/MS) for simultaneously quantifying nicotine, six nicotine metabolites, and two minor tobacco alkaloids in smokers' urine. This method enzymatically hydrolyzes conjugated nicotine (primarily glucuronides) and its metabolites. We then use acetone pretreatment to precipitate matrix components (endogenous proteins, salts, phospholipids, and exogenous enzyme) that may interfere with LC-MS/MS analysis. Subsequently, analytes (nicotine, cotinine, hydroxycotinine, norcotinine, nornicotine, cotinine N-oxide, nicotine 1′-N-oxide, anatabine, and anabasine) are chromatographically resolved within a cycle time of 13.5 minutes. The optimized assay produces linear responses across the analyte concentrations typically found in urine collected from daily smokers. Because matrix ion suppression may influence accuracy, we include a discussion of conventions employed in this procedure to minimize matrix interferences. Simplicity, low cost, low maintenance combined with high mean metabolite recovery (76–99%), specificity, accuracy (0–10% bias) and reproducibility (2–9% C.V.) make this method ideal for large high through-put studies.

## Introduction

Monitoring tobacco exposure by the use of urinary nicotine metabolite analysis has become an informative tool for evaluating the effectiveness of regulations intended to limit public exposure and tobacco distribution to minors [1]. Further, understanding the various metabolic processes involved in nicotine uptake and clearance may aid in optimizing and customizing cessation programs to improve their success rates [2], [3]. Urinary nicotine metabolite measurement may also be helpful for estimating the effectiveness of nicotine delivery systems (smokeless tobacco products) and nicotine replacement therapies (patch, gum, and inhalers). Urine nicotine metabolite profiles of tobacco users have been essential for the identification of variations in the metabolic processing of nicotine by selected population groups [4]–[10]. The minor tobacco alkaloids—anatabine and anabasine—are included in this method because they are detectable in tobacco smokers' urine and are not metabolites of nicotine. Because the use of nicotine gum, inhalers, or other nicotine delivery devices should not provide detectable amounts of these two alkaloids, the detection of anatabine and anabasine in the urine of participants in nicotine replacement therapy tobacco cessation programs can provide an indication of non-compliance [11], [12].

**Figure 1:**
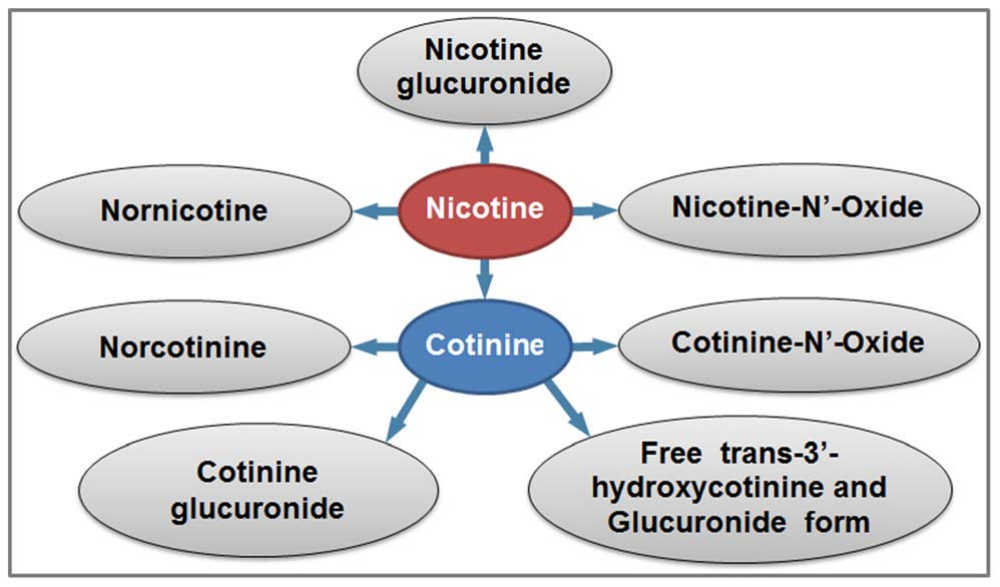
Nicotine metabolism. Following nicotine uptake in the body, nicotine is metabolized to six primary metabolites (cotinine, hydroxycotinine, norcotinine, nornicotine, cotinine oxide, and nicotine oxide). Nicotine, cotinine, and trans-3′-hydroxycotinine are subsequently glucuronidated at significant rates.

Following nicotine uptake in the body, nicotine is metabolized mainly via the P450 enzyme system to six primary metabolites:cotinine, hydroxycotinine, norcotinine, nornicotine, cotinine oxide, and nicotine oxide (**Figure 1**) [9]. In this paper we describe a LC-MS/MS method for determining concentrations of nicotine, these six nicotine metabolites, and two minor tobacco alkaloids—anabasine and anatabine—in urine. Nicotine and two metabolites—cotinine and hydroxycotinine—form substantial levels of conjugates (primarily glucuronides) that are excreted in the urine. We report these metabolites as “free” (non-conjugated forms) and “total” (sum of conjugated and non-conjugated forms). Individual measurement of the “free” and the “total” may provide useful metabolic rate information, such as the rate of individual or ethnic variations in glucuronidation [4]. Glucuronidation of toxins tends to increase their water solubility and accelerate their removal from the body through urinary excretion. For analysis of “total” analytes, we used *β*-glucuronidase to enzymatically remove the conjugated moiety. For measurement of “free” analytes, no enzymatic hydrolysis was performed prior to the acetone precipitation step and the subsequent LC-MS/MS analysis.

Several LC-MS/MS methods are available for determination of nicotine metabolites and tobacco related alkaloids. These pretreatment methods include liquid/liquid [13], [14], solid-phase extraction (SPE) [15], [16], acid precipitation [17], centrifugation, and filtration [18]. Although sensitive, these methods are time and labor intensive. Additionally, the use of SPE columns can be expensive. A protein/salt precipitation method that uses an organic solvent, such as acetonitrile or methanol is commonly applied. The method described here uses acetone precipitation pretreatment for depleting phospholipids, proteins, and salts from urine specimens prior to LC-MS/MS analysis. Advantages of the acetone pretreatment method include lower toxicity (vs. acetonitrile), lower cost (vs. SPE), and a lower labor requirement (vs. liquid/liquid). In this method acetone effectively removes exogenously added enzyme used for the hydrolysis of urinary glucuronides. Further, acetone evaporates more readily (vs. methanol, acetonitrile or water)—thus, facilitating sample concentration. In this method, we preferentially evaporate acetone from an acetone/urine mixture leaving residual urine for LC-MS/MS analysis. By avoiding complete urine evaporation we greatly enhanced the recovery of the volatile nicotine and nornicotine. Following the validation of this method, we analyzed urine specimens collected from 94 cigarette smokers.

## Materials and Methods

### Chemicals

Chemicals used in this work were obtained from the following sources: Acetone (Optima, A.C.S., Fisher Scientific, Cat. #A929SK-4); Acetonitrile (ACS/HPLC Certified Solvent, Honeywell B&J, Cat #AH015-4PC); Ammonium acetate (Fluka Analytical Cat. #73594-100G-F); Ammonium hydroxide (Certified A. C. S. PLUS, Fisher Scientific, Cat. #A669S-500); Hydrochloric acid (HCl) (Certified A.C.S. PLUS, Fisher Scientific, Cat. #A144-500); Methanol (HPLC/GC, Honeywell B&J, Cat. #230-4); Water (HPLC Tedia Company, Inc., Cat. #WS2211-001); *β*-Glucuronidase (type H-1, from *Helix pomatia*, Sigma, Cat. #G0751-2MU).

### Working solutions

#### Enzyme solution

An enzyme solution containing 10,000 units/mL of *β*-Glucuronidase (type H-1) was prepared by diluting the purified powder in 0.5 M ammonium acetate solution with pH of 5.1, adjusted using glacial acetic acid. If stored at 4–6°C, the enzyme retained sufficient activity for use in this procedure for up to 2 weeks.

#### Methanolic HCl solution

1% concentrated HCl in methanol (1 mL concentrated HCl in 100 mL volumetric flask, q.s. methanol).

#### Acidified HPLC water (pH∼3)

8 drops of concentrated HCl added to 4 liters of HPLC water.

#### Standard materials

Most standard materials were obtained from Toronto Research Chemicals, Ontario, Canada, including the following: Cotinine N-oxide (Catalog No. C725200); Cotinine N-oxide-methyl d3 (Catalog No. C725203); trans-3′-Hydroxycotinine (Catalog No. H92450); trans-3′-hydroxycotinine-methyl d3 (Catalog No. N427492); (R,S)-Norcotinine (Catalog No. N66200); (R,S)-Norcotinine-pyridyl d4 (Catalog No. N662002); (R,S)-Nornicotine (Catalog No. N757000); (R,S)-Nornicotine-pyridyl d4 (Catalog No. N757010); Nicotine-Methyl d3 (Catalog No N412425); (1′S, 2′S) Nicotine-1′-N-oxide (Catalog No N427500); (+/−)-trans- nicotinine-1′-N-oxide-methyl d3 (Catalog No. N427492); (R,S)-Anabasine (Catalog No. A637175); (R,S)-Anabasine-2,4,5,6-d4 (Catalog No. A637180); (R,S)-Anatabine (Catalog No. A637500); and (R,S)-Anatabine-2,4,5,6-d4 (Catalog No. A637505). Additional standards were obtained from Sigma-Aldrich, St. Louis, MO: (−)-Cotinine, (Catalog No. C-5923); (−)-Nicotine (Catalog No. N-3876) and from Cambridge Isotopes Laboratories, Andover, MA: Cotinine-Methyl d3 (Catalog No. DLM-1819).

#### Standard solutions

Standards were prepared and diluted using high pressure liquid chromatography (HPLC) grade water at pH ∼3. The acidic diluent retards the evaporative loss of nicotine and nornicotine from solution by keeping these analytes primarily in the ionized form. Twelve standards were prepared across the indicated concentration ranges. **Table 1** lists lists the reportable concentration range of the standard solutions (based on 200 µL sample). To avoid mass spectrometer detector saturation, each of the 12 standard solutions was diluted by a factor of 5 using acidic water and the channel electron multiplier (CEM) gain was set to provide a peak height response below 2.0×10^6^ when injecting the most concentrated standard solution. Following preparation the standards were dispensed into cryovials and stored at −70°C and a set was thawed before analysis. **Data S1** **. Standards preparation scheme.**

**Table 1:** Standard Concentrations and Reportable Ranges (sample volume 200 µL urine–1∶5 dilution factor).

Analyte	Internal Standard, ng	Standards Range, ng/mL	Reportable range*, ng/mL
Cotinine Oxide	100	1 to 2000	5 to 10,000
Nicotine 1′ Oxide	100	1 to 4000	5 to 20,000
Hydroxycotinine	100	2 to 8000	10 to 40,000
Norcotinine	100	0.4 to 2000	2 to 10,000
Cotinine	100	2 to 4000	10 to 20,000
Nornicotine	100	0.4 to 2000	2 to 10,000
Anatabine	50	0.4 to 200	2 to 1000
Anabasine	50	0.4 to 200	2 to 1000
Nicotine	100	2 to 4000	10 to 20,000

* Based on 200 µL sample (1 to 5 dilution factor).

#### Internal standard (ISTD) spiking solution

Stock ISTD solutions (100 mL) for each of the nine deuterated internal standards were gravimetrically prepared in volumetric flasks using acidified HPLC water as solvent. Dilute ISTD solutions (20 µg/mL) were prepared from each of the stock internal standards solutions, except anatabine-pyridyl-d4 and anabasine-pyridyl-d4, both prepared at 10 ug/mL. To prepare the ISTD spiking solution, 100 mL of each of the 9 dilute ISTD solutions was mixed in a 1 liter volumetric flask and diluted to a final volume of 1 liter with acidified HPLC water. The ISTD spiking solution was used for the preparation of each standard level (1–12). Following preparation of the 12 standard levels, the remaining ISTD spiking solution was dispensed into 2 mL cryovials and frozen at −20°C. A new cryovial containing ISTD spiking solution was thawed for each analytical run (∼24 samples). For sample preparation, 50 µL of ISTD spiking solution was added to each sample vial. The 50 µL ISTD spike contained 100 ng of each analyte, except for anatabine and anabasine—both 50 ng. Following preparation the internal standard spiking solution was dispensed into cryovials, stored at −70°C, and thawed before sample preparation.

#### Standard calibration

All 12 standard concentrations were analyzed and processed with each group of samples. Calibration was based on weighted regression analysis (1/X linear regression) by use of AB Sciex Analyst software (version 1.4.2). In all cases, an acceptable regression result required a Y-intercept less than 0.1 ng/mL and a correlation coefficient (*r*) that was greater than 0.98. Typically r values were >0.99.

### Instrumentation

LC-MS/MS analysis was performed using an AB Sciex (Framingham, MA) API 4000 triple quadrupole mass spectrometer with an electron ion spray interface and a Peak Scientific Ltd.(Scotland, UK), model NM20ZA, gas generator. The HPLC system consisted of a Shimadzu (Kyoto, Japan) SCL-10A system controller, two Shimadzu SC-10AD pumps, one Shimadzu DGU-14A degasser and an Agilent (Santa Clara, CA) 1200 series autosampler and column heater. A Beckman Coulter (Indianapolis, IN) model Allegra X-12R refrigerated centrifuge, a Thermo Fisher (Waltham, MA) Savant SpeedVac System SPD2010, and a Thermo Fisher Precision oscillating water bath were used during the sample preparation.

#### Mass spectrometer (MS)

The method employs an isotopic dilution reverse-phase LC-MS/MS method that uses electrospray ionization (ESI) and multiple reaction monitoring in positive ion mode at unit mass resolution to determine simultaneously the presence of nicotine, six nicotine metabolites and two tobacco components (anabasine and anatabine) in smokers' urine.

MS compound-dependent parameter settings—declustering potential (DP), entrance potential (EP), collision energy (CE), and collision cell exit potential (CXP)—were optimized using flow infusion. A syringe pump was used to introduce a solution containing a single native (not isotopically labeled) or labeled analyte. The optimal voltage settings were determined for each analyte and these optimal settings were used for the MS analysis, except for the cotinine and hydroxycotinine settings. **Data S2** **. Mass transitions.** The concentrations of these latter analytes in smokers' urine may greatly exceed the linear limits of the mass spectrometer if the optimized voltage settings are used. Therefore, the voltage settings (“CE” and “CXP”) for cotinine and hydroxycotinine were de-tuned to yield a lower response by the detector. De-tuning the optimal voltage settings for these two analytes allows quantification of both major and minor analytes in a single injection. **Data S3** **. De-tuning MS compound-specific parameters.**

Because CEM detector saturation is typically reached at a peak height of 2.0×10^6^, the CEM voltage setting was adjusted by injecting the response reference (hereafter “method blank”). The method blank is a sample in which water replaces both urine and enzyme and there is no acetone precipitation step. When injecting the method blank the CEM voltage setting was adjusted to produce a cotinine ISTD peak height of approximately 1.0×10^5^. This voltage setting provides a method linear response to approximately 20 times the ISTD (i.e., 1.0 e^5^ * 20 = 2.0 e^6^). Because the most concentrated cotinine standard is 20 times the cotinine ISTD concentration, a linear response is attained throughout the standard curve.

#### HPLC

Base-line chromatographic separation was obtained with a Phenomenex Gemini-NX, C18, 110A, HPLC column (4.6 mm×150 mm), 5 µm particle size (Part # 00F-4454-E0) and Gemini-NX pre-column (Part # AJO-8368). Two in-line filters were inserted prior to the analytical pre-column. The first filter uses an A-100X SS frit; the second filter uses an A-103X frit (Upchurch Scientific). The column oven was maintained at 40°C. Injection volume was 10 µL. Flow rate was 1.0 mL/min.

Mobile phase “A” was 6.5 mM ammonium acetate in HPLC grade water with pH adjusted to 10.5 using ammonium hydroxide; mobile phase “B” was 100% acetonitrile. A Shimadzu SCL-10AVP controller and two LC-10ADVP binary pumps were programed to deliver a timed gradient over 13.5 minutes, as outlined in **Table 2**.

**Table 2:** HPLC Gradient Elution Table.

Time, min.	Module	Event	Parameter
0.01	Controller	Solenoid Valve	BBB
0.02	Pumps	%B	3
1.00	Pumps	%B	3
9.00	Pumps	%B	30
10.50	Pumps	%B	30
10.51	Pumps	%B	100
11.20	Pumps	%B	100
11.21	Pumps	%B	3
13.50	Controller	Stop	

*Mobile phase A is 6.5 mM ammonium acetate, pH 10.5.

*Mobile phase B is 100% acetonitrile.

### Sample preparation

We report urine nicotine metabolites as “free” and “total”. For “total” sample preparation, we mixed 50 µL (100 ng) of isotopically labeled ISTD spiking solution, 100 µL of sample urine, 100 µL of HPLC water and 160 µl (1600 units) of enzyme solution. We then incubated the mixture at 37°C overnight (ca. 21 hours) [19]. If we were determining only “free” forms, we spiked 200 µL of urine (no water added) with 50 µL of ISTD solution, replaced the *β*-glucuronidase solution with an equivalent volume of water, and omitted the 37°C overnight water bath incubation. Then, we added 0.85 mL of cold acetone to each sample (“free” or “total”) to initiate the precipitation of salts, protein, and exogenous enzyme. After mixing, we refrigerated the samples at 4°C for greater than ten minutes; the resulting precipitate was removed by centrifugation (30 minutes at ∼3200 x g and 4°C). Immediately following centrifugation, we transferred a portion (∼380 µL) of the top urine/acetone solution to a LC injector vial (with 400 µL limited volume insert) containing 20 µL of methanolic HCl. Next, the acetone was removed by partial drying of urine/acetone solution in a Thermo Savant evaporator for 30 minutes. We directly injected the residual urine supernatant on the LC–MS/MS.

### Quality-control (QC) materials

QC pools were prepared by combining human urine from smokers and non-smokers in varying proportions to obtain high, medium or low analyte concentrations. The pools that resulted from the mixing process were spiked with additional minor analytes to achieve the desired analyte concentrations. Ten pools were prepared in support of this study. Final concentrations and initial quality control limits were determined using a minimum of twenty runs spread over about six weeks. The total cotinine concentrations of these pools ranged from 5 to 6000 ng/mL.

### Water blanks

A water blank (200 µL of HPLC water as sample) was included in each analytical run. Water blank calculated concentrations for each of the nine analytes, other than nornicotine and nicotine, were mostly not detectable. The upper limit assigned for rejecting runs on the basis of a high water blank concentration was set at 0.05 ng/mL for all analytes, except for nornicotine and nicotine. The nornicotine blank upper limit was assigned 0.1 ng/mL and the nicotine blank upper limit was assigned 0.2 ng/mL. These latter two analytes tend to be ubiquitous in the environment and attempting to achieve lower urine blanks was not feasible. However, for smoker urine assays, the blank levels maintained in this work are acceptable because the allowable blank upper limits were well below the lowest reported values for both nornicotine (2 ng/mL) and nicotine (10 ng/mL). All final results are blank-corrected by subtraction of the run blank analyte concentrations from the individual sample analyte concentrations.

### Representative chromatograms

**Figure 2:**
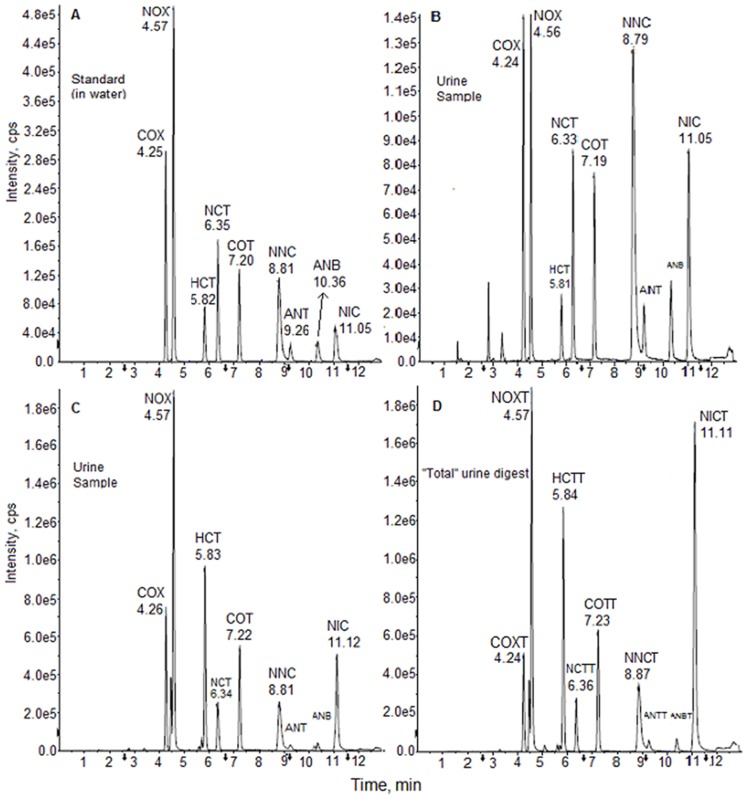
Representative chromatograms. (A) Standards Analysis (cotinine, 200 ng/mL); (B) Smoker Urine Sample (“free” cotinine, 14.1 ng/mL); (C) Smoker Urine Sample (“free” cotinine, 2767 ng/mL); (D) Smoker Urine Sample (“total” cotinine, 4195 ng/mL). Abbreviations: Cotinine-oxide (COX); Nicotine-oxide NOX); Hydroxycotine (HCT); Norcotinine (NCT); Cotinine (COT); Nornicotine (NNC); Anatabine (ANT); Anabasine (ANB); Nicotine (NIC). The fourth letter “T” in the abbreviations in Figure 2(D) represents the “total” concentrations for measured analytes.

**Figure 2(A)** provides a chromatogram from the analysis of a mid-range standard with a cotinine value of 200 ng/mL. **Figure 2(B)**, and **Figure 2(C)** provide two representative “free” analyses of a smoker's urine sample. The latter samples had a measured cotinine concentration of 14.1 ng/mL and 2.77 µg/mL. **Figure 2(D)** provides a chromatogram of an enzyme-treated sample representing a “total” metabolite analysis. The chromatograms reflect the typical peak resolution and baseline achieved by this method.

### Method accuracy and precision

A complete set of standards was analyzed and processed with each group of samples, and these standards were used to calculate the concentrations of each analyte, as described previously. The accuracy and precision of this method was examined by analyzing a series of spiked non-smokers' urine samples for each of the nine analytes. Five spiked urine pools (designated, TSV1 thru TSV5) were prepared by spiking non-smokers' urine with concentrated solutions of each analyte and analyzed 20 times over a period of six weeks. The accuracy and precision observed for this series is summarized in **Table 3**. For concentrations found in smoker's urine, results differed from the spiked pool target values by no more than 10% with less than 10% coefficient of variation (CV). **Data S4** **. Accuracy** **table 3**.

**Table 3:** Accuracy and Precision from the Repetitive Analysis of Five Fortified Urine Pools.

Pool	*Target, ng/mL (Observed Mean)* *	*STDEV (%CV)* *	*% Bias*	*Target, ng/mL (Observed Mean)* *	*STDEV (%CV)* *	*% Bias*	*Target, ng/mL (Observed Mean)* *	*STDEV (%CV)* *	*% Bias*
	**Cotinine N-oxide**			**Nicotine N-oxide**			**Hydroxycotinine**		
**TSV-1**	10	0.662	0.0	20	0.991	−0.36	20	2.510	0.5
	(10.0)	(5.5)		(19.9)	(4.9)		(20.97)	(12.0)	
**TSV-2**	40	0.878	0.14	40	1.569	0.001	160	13.02	−0.7
	(40.1)	(2.1)		(40.0)	(3.9)		(158.8)	(7.8)	
**TSV-3**	200	5.91	−0.10	400	17.52	0.1	800	44.08	1.4
	(199.8)	(2.9)		(400.3)	(4.4)		(811.1)	(5.4)	
**TSV-4**	100	3.64	−1.35	100	3.767	−1.4	2000	92.69	0.6
	(98.6)	(3.6)		(98.9)	(3.8)		(2012)	(4.6)	
**TSV-5**	400	13.0	−0.41	400	19.94	−0.4	4000	171.6	−0.4
	(398.4)	(3.3)		(399.9)	(5.0)		(3986)	(4.3)	
	**Nornicotine**			**Norcotinine**			**Cotinine**		
**TSV-1**	10	0.421	−4.3	10	0.965	−1.2	20	1.07	−1.2
	(9.57)	(4.4)		(9.88)	(9.5)		(19.8)	(4.0)	
**TSV-2**	40	1.15	−2.8	40	3.14	−0.2	160	4.69	−0.8
	(38.9)	(3.0)		(39.9)	(7.8)		(159)	(2.8)	
**TSV-3**	200	6.30	−1.5	200	14.5	−3.7	800	29.6	−1.1
	(197)	(3.2)		(193)	(7.5)		(792)	(3.7)	
**TSV-4**	100	3.31	−0.5	100	8.07	3.7	2000	70.5	−1.4
	(99.5)	(3.3)		(104)	(7.8)		(1972)	(3.6)	
**TSV-5**	400	10.7	−1.6	400	24.3	0.5	4000	119	−3.4
	(394)	(2.7)		(402)	(6.0)		(3866)	(3.1)	
	**Anatabine**			**Anabasine**			**Nicotine**		
**TSV-1**	0.5	0.127	−6.1	0.5	0.187	−8.2	20	0.656	−5.9
	(0.470)	(26.7)		(0.460)	(40.7)		(18.8)	(3.6)	
**TSV-2**	1.0	0.125	−4	1.0	0.158	0.33	40	1.05	−3.4
	(0.960)	(12.9)		(1.00)	(15.8)		(38.6)	(2.8)	
**TSV-3**	2.0	0.191	10	2.0	0.147	6.4	400	7.99	−3.3
	(2.21)	(8.6)		(2.13)	(6.9)		(387)	(2.1)	
**TSV-4**	5.0	0.316	10	5.0	0.288	5.5	1200	18.4	−4.1
	(5.51)	(5.7)		(5.28)	(5.5)		(1151)	(1.6)	
**TSV-5**	25	1.06	1.3	25	1.03	−0.87	2000	42.9	−4.0
	(25.3)	(4.2)		(24.8)	(4.1)		(1921)	(2.2)	

*Calculated values determined by analyzing each pool in 20 runs.

### Sample analyte stability

A urine pool was thawed and re-frozen eight times over two days and analyzed in comparison with samples maintained frozen at −70°C. No change was observed in the concentration of any of the nine analytes, or the conjugates. A separate study demonstrated that nicotine as well as the urine metabolites and conjugates are stable in urine at room temperature for at least 2 months when stored in sealed cryovials and protected from light. Storage at room temperature, with light exposure, resulted in some degradation of nornicotine and nicotine. **Data S5** **. RT light stability.** A degradation of 10–20% was evident for both nornicotine and nicotine when stored at room temperature in cryovials and exposed to indirect daylight over a seven week period. All analytes were stable during the seven week study when stored in the dark at 4°C or −20°C. Routinely, our urine control pools and study samples are maintained in low-temperature freezers at or below –20°C. These studies, and the continued results from our ongoing analysis of QC pools, demonstrate that the metabolites and alkaloids are stable during the analysis and storage conditions described in this text.

### Limit of detection (LOD)

The method detection limits for the analytes were defined as three times S_0_, where S_0_ is the estimate of the standard deviation at zero analyte concentration. The value of S_0_ is taken as the y-intercept of a linear regression of standard deviation versus the concentration (a minimum of four concentration levels of the analytes) as specified by Taylor [20] (**Table 4**). The LOD results were determined using twenty repetitive analyses of four native-spiked blank urine pools. For determination of “total” concentrations of each analyte, enzyme digestion was used in the pretreatment of the urine sample. For “free” analyte concentrations, no enzyme pretreatment was used. **Data S6** **. Precision and LOD determinations (free + total).** The LOD concentrations reported in **Table 4** are much lower that the lowest reportable analyte concentrations found in **Table 1**. This is because the **Table 1** concentrations are determined by the lowest concentration for each analyte in the standards set. In this method, reportable concentraion limits for each of the analytes in smokers' urine is the concentration of lowest standard for that analyte in the standard set, rather that the LOD of the analyte.

**Table 4:** Limits of Detection (LOD) for “free” and ”total” analytes.

Analyte	“Free”, LOD, ng/mL	“Total”, LOD, ng/mL
Cotinine Oxide	1.77	1.5
Nicotine 1′ Oxide	0.29	0.71
Hydroxycotinine	0.36	1.94
Norcotinine	0.48	0.62
Cotinine	1.4	3.53
Nornicotine	0.33	0.41
Anatabine	0.28	0.45
Anabasine	0.31	0.6
Nicotine	1.63	1.55

### Optimization of acetone volume

To estimate the volume of acetone that provides optimal recovery of the internal spiking solution with low ion suppression, the acetone volumes used in the precipitation step were varied. A control pool was analyzed using the procedure described here, except the volume of acetone used in the precipitation step was progressively increased from 0.5 mL to 5 mL. As previously reported for plasma, a ratio of urine to organic solvent of 1: 2 or 1: 2.5 is effective [21], [22]. The method described here uses a ratio of non-organic to organic of ∼1: 2.1. **Data S7** **. Acetone volume for precipitation 1.** **Data S8** **. Acetone volume for precipitation 2.**

### Optimization of internal standard concentrations

Because the paired native analytes and their labeled internal standards co-elute, they may compete for ionization in the MS source. If a very high native nicotine level in a sample suppresses the detector response of a much lower ISTD, an error in calculated concentration may occur. In order to maintain a constant response over the desired quantitation range, one must properly choose the amount of internal standard used for standards and for spiking samples [23]. In our method, the amount of labeled internal standard (100 ng) spiked into 200 µL of urine is about 20 to 50 times higher than the typical spike used for determining tobacco exposure in non-smokers. The use of a high concentration for the labeled ISTD improves the linear range of the highest standards and samples.

### Use of water-based standards versus urine-based standards

The use of water-based standards has several advantages over the use of urine-based standards. An important consideration is the consistency of urine between current and future standard preparations. Additional considerations include bacterial growth and decomposition.

To verify that water-based standards and urine based standards provide similar results when analyzing study samples, we generated a second standard curve by individually combining each of the 12 water-based standards (160 µL) with non-smoker urine (200 µL) to create a urine-based standard set which we then processed by use of the acetone precipitation procedure. We used a standard curve generated by these processed urine-based standards to quantify 18 urine pools (analyte enriched) in comparison to concentrations generated by use of water-based standards. Additionally, we generated a third standard curve to assure that treatment with the enzyme had minimal influence on the calculated analyte concentrations. To create an enzyme treated standard curve, the 12 water-based standards (200 µL) were each combined with enzyme solution (160 µL) and then processed by use of the acetone precipitation procedure. The results, as reported in **Table 5**, indicate that neither the urine nor the enzyme treatment contributed any discernable bias to the calculated concentrations of the 18 urine pools. **Data S9** **. Comparison of water, urine, and enzyme processed standards** **Table 5** **.**

**Table 5:** Comparison of standard curves generated by urine or enzyme standards to water standard curve.

	18 Pool Ranges	Water Standards		Urine Standards		Enzyme Standards	
Analyte	(lowest to highest), ng/mL	Slope	Intercept	Slope	Intercept	Slope	Intercept
Cotinine Oxide	1.4 to 556	1	0	1	−0.37	0.99	0
Nicotine Oxide	0.45 to 919	1	0	1.02	−1.95	1.01	−0.19
Hydroxycotinine	8.2 to 7158	1	0	0.96	−0.79	0.97	0
Norcotinine	0.26 to 386	1	0	1.04	−0.34	1.03	0
Cotinine	7.9 to 5931	1	0	1	−1.65	1	−0.26
Nornicotine	0.4 to 389	1	0	0.99	−0.41	1.02	0.08
Anatabine	0 to 42	1	0	1.02	0.11	0.98	0.2
Anabasine	0 to 27	1	0	1.01	−0.2	1	0.03
Nicotine	0 to 3253	1	0	1	−2.81	0.99	−0.3

### Sample recovery

The acetone precipitation step has an internal standard spike recovery of 76% to 99%. This was determined by preparing a set of six urine pools in two different ways. First, a set of six samples was prepared by adding the spiking internal standard in the first step of the analysis, as usual. In a second set of the same six samples, the spiking ISTD was added at the end—just before injection on the LC-MS/MS. When the two sets were injected into the LC-MS/MS, a comparison of the internal standards area counts indicated that a loss did not significantly occur during the precipitation step. However, sample recovery is adversely affected by both sample loss during acetone precipitation and by loss as a result of ion suppression during LC-MS/MS analysis. Therefore, we further examined the effect of matrix ion suppression.

### Ion suppression evaluation

To evaluate ion suppression in this method, two urine samples were analyzed at four levels of increasing dilution. If ion suppression is influencing the calculated analyte concentrations, these concentrations would be expected to increase or decrease with increasing dilution. The results observed are summarized in **Table 6** and demonstrate the good reproducibility of this method and the low influence of ion suppression on the calculated results. No substantial change in the analyte concentrations is indicated following increased dilutions (dilution 5 to dilution 50). All samples are single injections in a single run. **Data S10** **. Dilution influence on ion suppression** **Table 6** **.**

**Table 6:** Dilution influence on ion suppression. Abbreviations are according to Figure 2.

Sample (Volume)	Dilution factor	COXT	NOXT	HCTT	NCTT	COTT	NNCT	ANTT	ANBT	NICT
		ng/mL	ng/mL	ng/mL	ng/mL	ng/mL	ng/mL	ng/mL	ng/mL	ng/mL
Urine A	5	2.8	0	80.5	4.1	202	0.49	<LOD	0	8.5
(200 µl)										
Urine A	10	3.5	0	70.9	4.3	201	0.75	<LOD	0	8.2
(100 µl)										
Urine A	20	3.1	0	75.2	4.0	199	0.54	<LOD	0	8.0
(050 µl)										
Urine A	50	3.6	1.6	83	4.1	208	0.76	<LOD	0	8.7
(020 µl)										
Urine B	5	583	1001	5582	186	1483	151	44.9	20.1	3334
(200 µl)										
Urine B	10	576	991	5308	186	1540	154	41.6	21.3	3516
(100 µl)										
Urine B	20	542	977	5473	216	1586	157	38.7	21	3704
(050 µl)										
Urine B	50	563	1008	5548	207	1529	156	42	24	3786
(020 µl)										

### Ruggedness Test

Ruggedness testing was performed to access the influence of altering several pretreatment variables on the calculated analyte concentrations. The variables altered include pH of enzyme buffer solution, enzyme hydrolysis time, and the concentration of enzyme. The range of variations evaluated had no substantial effect on the calculated concentrations of the analytes. The results are displayed in the supplemental data files. **Data S11** **. Ruggedness testing.**

### Ethics statement

This study was reviewed and IRB approved by the Institutional Review Board at the Centers for Disease Control (protocol #6358) and complied with all national and international guidelines on research involving human subjects. All study participants provided written/witnessed consent using a preapproved consent form prior to donation of specimens. The urine samples tested were from 94 smokers who reported routinely smoking an average of 19 cigarettes/day.

## Results and Discussion

### Nicotine metabolite profile results

We present here a few initial observations relating to the distribution of the nicotine metabolites in a subset of samples to which we applied the method.

**Table 7** reports the molar percent of urinary nicotine metabolites found using this method to the molar percent compiled from previously reported 24-hour urine studies using various other methods [24]. The ratios are similar. Because the half-life of nicotine in serum is about 2 hours, an increase in nicotine percent in our daytime, spot-collected samples would be expected. A daytime collection during active smoking would yield a higher nicotine level than a sample collected following several hours of sleep, as would occur in a 24 hour urine studies. **Data S12** **. Molar % of urine metabolites in smokers** **Table 7** **.**

**Table 7:** The molar percent each analyte contributed to the combined concentrations of nicotine and 6 nicotine metabolites in this study (n  =  94).

	COXT	NOXT	HCTT	NCTT	COTT	NNCT	NICT
This Method %	4.0	7.8	38.8	1.3	26.4	1.1	20.6
(SD)	(1.3)	(7.9)	(20.7)	(0.3)	(9.6)	(0.4)	(14.2)
Other methods (compiled*) %	2–5	4–7	40–49	1–2	22–32	0.4–0.8	11–15

*compiled 24 hour studies adapted from Hukkanen et al. (2005) [29].

### Anatabine and anabasine as a tobacco exposure indicators

We evaluated the reliability of urinary anatabine and anabasine measurements as indicators of active smoking behavior. Anatabine and anabasine, two tobacco alkaloids that are not metabolites of nicotine, are included in this method because they have been measured in smoking cessation programs to validate non-smoking compliance [11]. Their presence in the urine of program participants is an indication of tobacco exposure. Recent tobacco use is indicated by urine concentrations of either alkaloid above the level of 2 ng/mL [11]. In the group of 94 smokers' urine samples, “total” anabasine and “total” anatabine were evaluated for their usefulness in validating smoking status. Of the 94 smoker urine samples, 94% had anatabine levels greater than 2 ng/mL. Only 74% of smokers had anabasine levels above 2 ng/mL. In addition, 94% had either anatabine or anabasine levels greater than 2 ng/mL. The mean anatabine level of all smokers was 15.2 ng/mL (range, 1.2 to 62.3 ng/mL). For anabasine the mean was 6.12 ng/mL (range, <LOD (0.60) to 30.0 ng/mL). For our group of heavy smokers, these results indicate that a cut-off of 2 ng/mL for anatabine is a more reliable indicator of smoking status than anabasine alone and is as reliable an indicator as combining the smoking status results of both anabasine and anatabine. These cut-off points may be further improved by including a measure of variable hydration such as creatinine correction. **Data 13. Anatabine and anabasine levels in 94 smokers.**

### Acetone as a precipitating agent

As a precipitating agent, acetone and acetonitrile are more effective in removing protein than is methanol [25]. Although the effectiveness of removing protein and salt by acetonitrile or acetone is similar [25], acetone may be more effective in removing phospholipids because most human endogenous phospholipids are insoluble in acetone, and acetone is sometimes used in harvesting phospholipids for quantification in body fluids [26]–[29]. Removal of phospholipids, proteins, and salts is beneficial in several ways. The removal reduces LC maintenance (plugged tubing and frits), extends column life, and eliminates some potential chromatographic interferences and ion suppressing components.

### Matrix ion suppression optimization

Matrix ion suppression should be considered as a possible source of inaccuracy when one is validating a LC-MS/MS method. Ion suppression occurs as a result of the presence of substances in the sample matrix that interfere with ion transmission by inhibiting analyte ionization and subsequent passage of ions through the MS orifice. During the development of a LC-MS/MS method, ion suppression may be addressed by application of practices that reduce suppression. For simplicity, the practices considered for limiting suppression in developing this method are briefly discussed under three headings: sample (preparation, dilution factor, ISTD); HPLC (mobile phase, injection volume, gradient elution, and column rinsing); and MS (ionization mode). Then, we follow with a description of the technique we used to monitor individual sample injections for excessive ion suppression.

### Ion suppression—sample

#### Sample preparation

Ion suppression may occur in any LC-MS/MS analysis following diverse types of sample preparation. Although ion suppression following direct injection or a “dilute and shoot” (solvent dilution and direct LC-MS/MS injection) or a “crash and burn” (protein precipitation and fast gradient LC-MS/MS run) method is more frequently present [30], it may also be observed during sample preparation by SPE or liquid/liquid extraction [31]. The concentration steps used in SPE or liquid/liquid sample preparations are a valuable means of increasing sensitivity and lowering the LOD, but they may also magnify matrix ion suppression by concentrating the suppressing components [32]. In our method, depletion of phospholipids, proteins, and salts by acetone precipitation eliminates many potential ion suppressing agents without use of a strong concentrating step, which is a suitable approach for smokers' urine samples.

#### Sample dilution factor

Ion suppression is generally decreased by increasing the dilution (addition of water, mobile phase, solvent) [33]. However, when deciding on an appropriate dilution, one must consider that excessive dilution can result in reduced sensitivity, as well as a higher LOD and coefficient of variation. In this assay, the water addition during the acetone precipitation step reduced suppression while maintaining suitable sensitivity for the application.

#### Sample ISTD

Although the influence of sample matrix on the calculated concentration of an analyte is greatly diminished by the use of isotopically labeled internal standards, ion suppression can either increase or decrease calculated values as well [34], [35]. In this method, isotopically labeled internal standards were used for all analytes. Because the native analyte and the labeled internal standard co-elute, they can suppress the response of each other. In order to maintain a constant response over the desired quantification range, one must properly choose the amount of internal standard used for spiking the sample [23]. In our method, the amount of labeled internal standard (100 ng) spiked into 200 µL of urine is about 20 to 50 times higher than the optimal spike concentration used for determining tobacco exposure in non-smokers. The use of a higher ISTD is beneficial because it provides more linearity at the high end of the standard curve and thus increases the range of reportable results.

### Ion suppression—HPLC

#### HPLC mobile phase

Although the use of an alkaline mobile phase in MS negative ion mode generally augments negative ion generation and improves sensitivity, the use of alkaline mobile phase in positive ion mode ionization may also provide improvement in sensitivity. The use of alkaline mobile phase may reduce chemical noise, thereby improving the signal-to-noise ratio—thus resulting in a lower LOD [36], [37]. Modifying the pH of the mobile phase has little effect on the elution time of phospholipids [38]; however, changing the mobile phase pH may alter the polarity of some analytes, thus altering elution times on a reverse phase HPLC column [39]. In the method described here, the use of alkaline mobile phase improves the resolution of some analytes (nicotine, nornicotine) since they elute later in the chromatogram because of polarity shifts. There is no significant loss in analyte response, and reduced sign-to-noise ratio improves LOD and facilitates automated integration of analyte peaks.

#### HPLC injection volume

Injection volumes should be low, thus maintaining chromatographic resolution and reducing the amount of suppressing agents competing with the analyte for ionization in the instrument source [40], [41]. Our method uses a 10 µL injection volume.

#### HPLC gradient

Analytes should be retained and well resolved by the LC column selected for the liquid chromatographic separation. Analytes eluting early (near the column void) are commonly affected by ion suppression [42] [43]. However, ion suppression may be observed at any elution time in a chromatographic separation that uses LC-MS/MS. The “crash and burn” methods in which the sample is precipitated with an organic solvent and then injected with little or no chromatographic resolution of the constituents are more susceptible to calculation inaccuracies because of ion suppression. In our method, no analytes elute near the column void and all are chromatographically base-line resolved.

#### HPLC column rinsing

Rinsing the LC column with a high concentration of the eluting mobile phase will help avoid carrying any residual suppressing components into the next injection cycle. In this urine method, at a flow rate of 1.0 mL/minute, a 0.5 minute column rinse using 100% acetonitrile followed by a 2.3 minute column regeneration to the initial gradient conditions was completed following each sample run. Our method analyzes urine samples; however, for some biological matrixes (blood, tissue extracts), an extended rinsing cycle with 100% eluting organic phase may be required.

### MS ionization mode

Two modes of ionization are available on the Sciex API 4000, ESI or atmospheric pressure chemical ionization (APCI). Although APCI is usually less susceptible to ion suppression than ESI [42], [44], [45], the use of APCI is not always applicable. ESI is used in our method because one of the analytes (nicotine 1′-N-oxide) is heat labile, and quantification by APCI is not possible.

### Ion suppression monitoring

Because ion suppression may occur at any point in a chromatographic elution and may vary among samples, it is desirable to have a dynamic evaluation of the ion suppression for each MS transition with each sample injection. In our method, to assess ion suppression in each sample, we compared the area of the internal standard recovered following LC-MS/MS injection of a urine sample to the internal standard recovered following the method blank injection [46]. As previously discussed, the method blank is a sample in which the urine is replaced by water and the acetone precipitation step is deleted. In our method, the “method blank” contains: 50 µL internal standard spiking solution, 360 µL water (replaces urine and enzyme), and 20 µL methanolic HCl. Because matrix urine components are absent, ion suppression is very low in the “method blank”. This comparison (sample ISTD to blank ISTD counts) does not provide an exact representation of the ion-suppression, but it does allow for detection of excessive suppression that may adversely affect calculated concentrations. Hoofnagle [47] determined that an imprecision of 25% was not reached unless the internal standard count was suppressed more than 90%. For our method, an ion suppression of greater than 50% flags the analysis to be repeated at a higher dilution. Diluting the sample reduces the ion suppression–a concept used in “dilute and shoot” methods.

Monitoring a second transition for the native analyte being quantified and establishing a nominal ratio between the areas of the two transitions may aid in detecting the presence of any interfering compound or of severe ion suppression. In our method, we monitor two transitions for each analyte and we monitor the calculated ratio (confirm/quant ratio) to flag samples requiring repeat analysis.

### Summary of method validation

Our validation of this method for nicotine, six nicotine metabolites, anatabine and anabasine has demonstrated its accuracy over the wide concentration range of smokers' urine specimens. Prior to the development of this method, multiple dilutions of smokers' urine samples were required because of the limited linear range of the API 4000 mass spectrometer. As described, the range of reportable analyte concentrations was extended by diluting the standards to achieve linear responses at the highest standard levels (ranges, **Table 1**), by the use of appropriate concentrations of isotopically labeled internal standards for each analyte, and by de-tuning the API 4000 instrument parameters (“CE” and “CXP”) for the two most abundant metabolites, cotinine and hydroxycotinine.

We verified the accuracy and precision of the method by use of spiked non-smoker urine pools (**Table 3**) and demonstrated the stability of the analytes during storage and analysis. The acetone precipitation pretreatment provides high mean metabolite recovery (76–99%) and analysis of the residual urine supernatant by the API 4000 produced LODs ranging from 0.41 to 3.53 ng/mL (**Table 4**). We employed progressive dilution of two smoker's urine samples to evaluate inaccuracies introduced by excessive ion suppression (**Table 5**). We confirmed that the use of water-based and urine-based standards both provide similar regression curves and provide calculated concentration differences of less than +/−3% when used to analyze 18 random smokers' urine samples (**Table 6**). We obtained chromatographically base-line resolution of all analytes (**Figure 2, A-D**) to reduce the possibility of interferences and ion suppression in random non-conforming samples.

## Conclusion

Acetone precipitation prior to LC-MS/MS analysis of urine components is a simple, accurate, inexpensive, relatively non-toxic sample preparation method. Although acetone precipitation is not commonly employed in the LC-MS/MS analysis of non-precipitated residual small molecules in a urine matrix, the removal of phospholipids, protein, and salts by acetone precipitation is effective in producing a sample that is highly compatible with analysis by LC-MS/MS. Because of acetone's high volatility, the acetone can be evaporated from the urine/acetone supernatant following precipitation, leaving the residual urine supernatant for injection. Being able to avoid complete dry-down of the urine sample improves recovery of the volatile analytes, especially nicotine and nornicotine. Simplicity, low-cost, low maintenance combined with high mean metabolite recovery (76–99%), specificity, accuracy (0-10% bias) and reproducibility (2-9% C.V.) make this method ideal for large high through-put studies. Acetone precipitation will likely be employed frequently in future analysis of urine small molecular components by LC-MS/MS. We look forward to exploring other applications.

## Supporting Information

Data S1 **Standards preparation scheme.**
(DOCX)

Data S2 **Mass transitions.**
(DOCX)

Data S3 **De-tuning MS compound-specific parameters.**
(DOCX)

Data S4 **Accuracy** **Table 3** **.**
(XLS)

Data S5 **RT light stability.**
(XLS)

Data S6 **Precision and LOD determinations (free + total).**
(XLS)

Data S7 **Acetone volume for precipitation 1.**
(XLS)

Data S8 **Acetone volume for precipitation 2.**
(DOCX)

Data S9 **Comparison of water, urine, and enzyme processed standards** **Table 5** **.**
(DOCX)

Data S10 **Dilution influence on ion suppression** **Table 6** **.**
(DOCX)

Data S11 **Ruggedness testing.**
(DOCX)

Data S12 **Molar % of urine metabolites in smokers** **Table 7** **.**
(ODS)

Data S13 **Anatabine and anabasine levels in 94 smokers.**
(XLS)
