# Supplementary material for: Validation of a LC-MS/MS Method for Quantifying Urinary Nicotine, Six Nicotine Metabolites and the Minor Tobacco Alkaloids—Anatabine and Anabasine—in Smokers' Urine
Source: PLoS One. 2014 Jul 11;9(7):e101816. doi: 10.1371/journal.pone.0101816 (PMC4094486; doi:10.1371/journal.pone.0101816)
Supplement: Data S1 — Standards preparation scheme. (DOCX) [file pone.0101816.s001.docx]

|  |  |  | **COTININE-N-OXIDE** | | | **MW = 192.22** | |  |  |
| --- | --- | --- | --- | --- | --- | --- | --- | --- | --- |
|  |  |  |  |  |  |  |  |  |  |
| **Native Stocks:** | |  |  |  |  |  |  |  |  |
| **Stock CXO Prep:** | | **117.6** | ug/mL |  |  |  |  |  |  |
|  | **Weight, mg/mL** | **% Purity** | **Corrected weight** | **Flask Vol., mL** | **Conc., mg/mL** |  |  |  |  |
|  | 12 | 0.98 | 11.76 | 100 | 0.1176 |  |  |  |  |
| **Stock CX1 Prep:** | | **100** | ug/mL |  |  |  |  |  |  |
|  | Vol CX0 | Vol., mL |  |  |  |  |  |  |  |
|  | 85.0340136 | 100 |  |  |  |  |  |  |  |
| **Stock CX2 Prep:** | | **20** | ug/mL |  |  |  |  |  |  |
|  | Vol CX1 | Vol., mL |  |  |  |  |  |  |  |
|  | 20 | 100 |  |  |  |  |  |  |  |
| **Stock CX3 Prep:** | | **0.2** | ug/mL |  |  |  |  |  |  |
|  | Vol CX2 | Vol., mL |  |  |  |  |  |  |  |
|  | 1 | 100 |  |  |  |  |  |  |  |
|  |  |  |  |  |  |  |  |  |  |
| **Labeled Stocks:** | |  | Cot-N-oxide-methyl-d3 |  |  |  |  |  |  |
|  |  |  |  |  |  |  |  |  |  |
| **Stock CXDO Prep:** | | **115.8018** | ug/mL |  |  |  |  |  |  |
|  | **Weight, mg/mL** | **% Purity** | **Corrected weight** | **Flask Vol., mL** | **Conc., mg/mL** | **Mol. Wt.Native** | **Mol. Wt. Labeled** | **Mol.wt adjust** |  |
|  | 12 | 0.98 | 11.76 | 100 | 0.1176 | 193.2 | 196.2 | 0.984709 |  |
| **Stock CXDA Prep:** | | **20** | ug/mL |  |  |  | 2 | ug/mL |  |
|  | **Vol CXDO** | **Vol., mL** | combine all ISTD solns to a volume of 5 liter | | | | **Vol., mL** |  |  |
|  | 86.3544175 | 500 |  |  |  |  | 5000 |  |  |
|  |  |  |  |  |  |  |  |  |  |
| **level** | **Native, ng / mL** | **Final Vol.** | **Native Total ng** | **Native Stock, ug/mL** | **Vol. of Native Stock, mL** | **ISTD Vol CXDA, mL** | **ISTD Vol Combined ISTD, mL** | **STD ISTD conc. ng/mL** | **spike ISTD, ng/ 50uL** |
|  |  | Use 500 mL |  | 0.2 |  |  |  |  |  |
| **1** | 0 | 100 | 0 |  | **0** | 5 | 50 | 1000 | 100 |
| **2** | 0.1 | 100 | 10 | CX3 | **0.05** | 5 | 50 | 1000 | 100 |
| **3** | 0.5 | 100 | 50 | CX3 | **0.25** | 5 | 50 | 1000 | 100 |
| **4** | 1 | 100 | 100 | CX3 | **0.5** | 5 | 50 | 1000 | 100 |
| **5** | 2 | 100 | 200 | CX3 | **1** | 5 | 50 | 1000 | 100 |
| **6** | 10 | 100 | 1000 | CX3 | **5** | 5 | 50 | 1000 | 100 |
|  |  |  |  | 20 | 6.8 |  |  |  |  |
| **7** | 20 | 100 | 2000 | CX2 | **0.1** | 5 | 50 | 1000 | 100 |
| **8** | 50 | 100 | 5000 | CX2 | **0.25** | 5 | 50 | 1000 | 100 |
| **9** | 100 | 100 | 10000 | CX2 | **0.5** | 5 | 50 | 1000 | 100 |
| **10** | 200 | 100 | 20000 | CX2 | **1** | 5 | 50 | 1000 | 100 |
| **11** | 400 | 100 | 40000 | CX2 | **2** | 5 | 50 | 1000 | 100 |
| **12** | 600 | 100 | 60000 | CX2 | **3** | 5 | 50 | 1000 | 100 |
|  |  |  |  | 100 | 6.85 |  |  |  |  |
| **13** | 1000 | 100 | 100000 | CX1 | **1** | 5 | 50 | 1000 | 100 |
| **14** | 2000 | 100 | 200000 | CX1 | **2** | 5 | 50 | 1000 | 100 |
| **15** | 3000 | 100 | 300000 | CX1 | **3** | 5 | 50 | 1000 | 100 |
| **16** | 4000 | 100 | 400000 | CX1 | **4** | 5 | 50 | 1000 | 100 |
|  |  |  |  |  | 10 | 80 |  |  |  |
|  | Spiking Solution Prep: |  |  |  |  |  |  |  |  |
|  | **Final Vol.(mL) of combined ISTD** | **Spike (ng)** | **Spike Vol.(uL) (mL/20)** | **ng in 5.0 L combined ISTD** | **Conc. of ISTD in combined ISTD, ng/mL** | **mL of combined ISTD needed for 100ng/ 1.0 mL of urine** |  |  |  |
|  | 5000.00 | 100 | 50 | 10000000 | 2000 | **0.05** |  |  |  |

|  |  |  | **NICOTINE-1’-OXIDE** | | | **MW = 178.19** | |  |  |
| --- | --- | --- | --- | --- | --- | --- | --- | --- | --- |
|  |  |  |  |  |  |  |  |  |  |
| **Native Stocks:** |  |  |  |  |  |  |  |  |  |
| **Stock NXO Prep:** | | **598.486** | ug/mL |  |  |  |  |  |  |
|  | **Weight, mg/mL** | **% Purity** | **Corrected weight** | **Flask Vol., mL** | **Conc., mg/mL** |  |  |  |  |
|  | 61.07 | 0.98 | 59.8486 | 100 | 0.598486 |  |  |  |  |
| **Stock NX1 Prep:** | | **200** | ug/mL |  |  |  |  |  |  |
|  | Vol NXO | Vol., mL |  |  |  |  |  |  |  |
|  | 33.41766 | 100 |  |  |  |  |  |  |  |
| **Stock NX2 Prep:** | | **50** | ug/mL |  |  |  |  |  |  |
|  | Vol NX1 | Vol., mL |  |  |  |  |  |  |  |
|  | 25 | 100 |  |  |  |  |  |  |  |
| **Stock NX3 Prep:** | | **0.5** | ug/mL |  |  |  |  |  |  |
|  | Vol NX2 | Vol., mL |  |  |  |  |  |  |  |
|  | 1 | 100 |  |  |  |  |  |  |  |
|  |  |  |  |  |  |  |  |  |  |
| **Labeled Stocks:** |  |  | Nic-N-oxide-methyl-d3 |  |  |  |  |  |  |
|  |  |  |  |  |  |  |  |  |  |
| **Stock NXDO Prep:** | | **115.6626** | ug/mL |  |  |  |  |  |  |
|  | **Weight, mg/mL** | **% Purity** | **Corrected weight** | **Flask Vol., mL** | **Conc., mg/mL** | **Mol. Wt.Native** | **Mol. Wt. Labeled** | **Mol.wt adjust** |  |
|  | 12 | 0.98 | 11.76 | 100 | 0.1176 | 179.1 | 182.1 | 0.983526 |  |
| **Stock NXDA Prep:** | | **20** | ug/mL |  |  |  | 2 | ug/mL |  |
|  | **Vol. NXD0** | **Vol., mL** | combine all ISTD solns to a volume of 5 liter | | | | **Vol., mL** |  |  |
|  | 86.45837 | 500 |  |  |  |  | 5000 |  |  |
|  |  |  |  |  |  |  |  |  |  |
| **level** | **Native, ng / mL** | **Final Vol.** | **Native Total ng** | **Native Stock, ug/mL** | **Vol. of Native Stock, mL** | **ISTD Vol NXDA, mL** | **ISTD Vol Combined ISTD, mL** | **STD ISTD conc. ng/mL** | **spike ISTD, ng/ 50uL** |
|  |  | Use 500 mL |  | 0.5 |  | Use 50 mL |  |  |  |
| **1** | 0 | 100 | 0 |  | **0** | 5 | 50 | 1000 | 100 |
| **2** | 0.1 | 100 | 10 | NX3 | **0.02** | 5 | 50 | 1000 | 100 |
| **3** | 0.5 | 100 | 50 | NX3 | **0.1** | 5 | 50 | 1000 | 100 |
| **4** | 1 | 100 | 100 | NX3 | **0.2** | 5 | 50 | 1000 | 100 |
| **5** | 2 | 100 | 200 | NX3 | **0.4** | 5 | 50 | 1000 | 100 |
| **6** | 10 | 100 | 1000 | NX3 | **2** | 5 | 50 | 1000 | 100 |
|  |  |  |  | 50 | **2.72** |  |  |  |  |
| **7** | 20 | 100 | 2000 | NX2 | **0.04** | 5 | 50 | 1000 | 100 |
| **8** | 50 | 100 | 5000 | NX2 | **0.1** | 5 | 50 | 1000 | 100 |
| **9** | 100 | 100 | 10000 | NX2 | **0.2** | 5 | 50 | 1000 | 100 |
| **10** | 500 | 100 | 50000 | NX2 | **1** | 5 | 50 | 1000 | 100 |
| **11** | 1000 | 100 | 100000 | NX2 | **2** | 5 | 50 | 1000 | 100 |
| **12** | 2000 | 100 | 200000 | NX2 | **4** | 5 | 50 | 25000 | 100 |
|  |  |  |  | 200 | **3.34** |  |  |  |  |
| **13** | 4000 | 100 | 400000 | NX1 | **2** | 5 | 50 | 1000 | 100 |
| **14** | 6000 | 100 | 600000 | NX1 | **3** | 5 | 50 | 1000 | 100 |
| **15** | 8000 | 100 | 800000 | NX1 | **4** | 5 | 50 | 1000 | 100 |
| **16** | 10000 | 100 | 1000000 | NX1 | **5** | 5 | 50 | 1000 | 100 |
|  |  |  |  |  | 14 | 80 |  |  |  |
|  | Spiking Solution Prep: |  |  |  |  |  |  |  |  |
|  | **Final Vol.(mL) of combined ISTD** | **Spike (ng)** | **Spike Vol.(uL) (mL/20)** | **ng in 5.0 L combined ISTD** | **Conc. of ISTD in combined ISTD, ng/mL** | **mL of combined ISTD needed for 100ng/ 1.0 mL of urine** |  |  |  |
|  | 5000.00 | 100 | 50 | 10000000 | 2000 | **0.05** |  |  |  |

|  |  |  | **3-OH Cot** |  |  | **MW = 192.20** |  |  |  |
| --- | --- | --- | --- | --- | --- | --- | --- | --- | --- |
|  |  |  |  |  |  |  |  |  |  |
| **Native Stocks:** |  |  |  |  |  |  |  |  |  |
| **Stock HCO Prep:** | | **882** | ug/mL |  |  |  |  |  |  |
|  | **Weight, mg/mL** | **% Purity** | **Corrected weight** | **Flask Vol., mL** | **Conc., mg/mL** |  |  |  |  |
|  | 90 | 0.98 | 88.2 | 100 | 0.882 |  |  |  |  |
| **Stock HC1 Prep:** | | **400** | ug/mL |  |  |  |  |  |  |
|  | Vol HCO | Vol., mL |  |  |  |  |  |  |  |
|  | 45.3515 | 100 |  |  |  |  |  |  |  |
| **Stock HC2 Prep:** | | **20** | ug/mL |  |  |  |  |  |  |
|  | Vol HC1 | Vol., mL |  |  |  |  |  |  |  |
|  | 5 | 100 |  |  |  |  |  |  |  |
| **Stock HC3 Prep:** | | **0.2** | ug/mL |  |  |  |  |  |  |
|  | Vol HC2 | Vol., mL |  |  |  |  |  |  |  |
|  | 1 | 100 |  |  |  |  |  |  |  |
|  |  |  |  |  |  |  |  |  |  |
| **Labeled Stocks:** |  |  | 3-hydroxycotinine-methyl-d3 |  |  |  |  |  |  |
|  |  |  |  |  |  |  |  |  |  |
| **Stock HCD0 Prep:** | | **115.802** | ug/mL |  |  |  |  |  |  |
|  | **Weight, mg/mL** | **% Purity** | **Corrected weight** | **Flask Vol., mL** | **Conc., mg/mL** | **Mol. Wt.Native** | **Mol. Wt. Labeled** | **Mol.wt adjust** |  |
|  | 12 | 0.98 | 11.76 | 100 | 0.1176 | 193.2 | 196.2 | 0.9847 |  |
| **Stock HCDA Prep:** | | **20** | ug/mL |  |  |  | 2 | ug/mL |  |
|  | **Vol HCD0** | **Vol., mL** | combine all ISTD solns to a volume of 5 liter | | | | **Vol., mL** |  |  |
|  | 86.3544 | 500 |  |  |  |  | 5000 |  |  |
|  |  |  |  |  |  |  |  |  |  |
| **level** | **Native, ng / mL** | **Final Vol.** | **Native Total ng** | **Native Stock, ug/mL** | **Vol. of Native Stock, mL** | **ISTD Vol HCDA, mL** | **ISTD Vol Combined ISTD, mL** | **STD ISTD conc. ng/mL** | **spike ISTD, ng/ 50uL** |
|  |  | Use 500 mL |  | 0.2 |  | Use 50 mL |  |  |  |
| **1** | 0 | 100 | 0 |  | **0** | 5 | 50 | 1000 | 100 |
| **2** | 0.1 | 100 | 10 | HC3 | **0.05** | 5 | 50 | 1000 | 100 |
| **3** | 0.2 | 100 | 20 | HC3 | **0.1** | 5 | 50 | 1000 | 100 |
| **4** | 0.4 | 100 | 40 | HC3 | **0.2** | 5 | 50 | 1000 | 100 |
| **5** | 1 | 100 | 100 | HC3 | **0.5** | 5 | 50 | 1000 | 100 |
| **6** | 4 | 100 | 400 | HC3 | **2** | 5 | 50 | 1000 | 100 |
|  |  |  |  | 20 | 2.85 |  |  |  |  |
| **7** | 10 | 100 | 1000 | HC2 | **0.05** | 5 | 50 | 1000 | 100 |
| **8** | 40 | 100 | 4000 | HC2 | **0.2** | 5 | 50 | 1000 | 100 |
| **9** | 100 | 100 | 10000 | HC2 | **0.5** | 5 | 50 | 1000 | 100 |
| **10** | 300 | 100 | 30000 | HC2 | **1.5** | 5 | 50 | 1000 | 100 |
| **11** | 600 | 100 | 60000 | HC2 | **3** | 5 | 50 | 1000 | 100 |
| **12** | 1000 | 100 | 100000 | HC2 | **5** | 5 | 50 | 1000 | 100 |
|  |  |  |  | 400 | 10.25 |  |  |  |  |
| **13** | 2000 | 100 | 200000 | HC1 | **0.5** | 5 | 50 | 1000 | 100 |
| **14** | 4000 | 100 | 400000 | HC1 | **1** | 5 | 50 | 1000 | 100 |
| **15** | 8000 | 100 | 800000 | HC1 | **2** | 5 | 50 | 1000 | 100 |
| **16** | 12000 | 100 | 1200000 | HC1 | **3** | 5 | 50 | 1000 | 100 |
|  |  |  |  |  | 6.5 | 80 |  |  |  |
|  | Spiking Solution Prep: |  |  |  |  |  |  |  |  |
|  | **Final Vol.(mL) of combined ISTD** | **Spike (ng)** | **Spike Vol.(uL) (mL/20)** | **ng in 5.0 L combined ISTD** | **Conc. of ISTD in combined ISTD, ng/mL** | **mL of combined ISTD needed for 100ng/ 1.0 mL of urine** |  |  |  |
|  | 5000.00 | 100 | 50 | 10000000 | 2000 | **0.05** |  |  |  |

|  |  |  | **NORCOTININE** | | | **MW = 162.19** | |  |  |
| --- | --- | --- | --- | --- | --- | --- | --- | --- | --- |
|  |  |  |  |  |  |  |  |  |  |
| **Native Stocks:** | |  |  |  |  |  |  |  |  |
| **Stock NCO Prep:** | | **98** | ug/mL |  |  |  |  |  |  |
|  | **Weight, mg/mL** | **% Purity** | **Corrected weight** | **Flask Vol., mL** | **Conc., mg/mL** |  |  |  |  |
|  | 10 | 0.98 | 9.8 | 100 | 0.098 |  |  |  |  |
| **Stock NC1 Prep:** | | **50** | ug/mL |  |  |  |  |  |  |
|  | Vol NCO | Vol., mL |  |  |  |  |  |  |  |
|  | 51.02041 | 100 |  |  |  |  |  |  |  |
| **Stock NC2 Prep:** | | **2** | ug/mL |  |  |  |  |  |  |
|  | Vol NC1 | Vol., mL |  |  |  |  |  |  |  |
|  | 4 | 100 |  |  |  |  |  |  |  |
| **Stock NC3 Prep:** | | **0.02** | ug/mL |  |  |  |  |  |  |
|  | Vol NC2 | Vol., mL |  |  |  |  |  |  |  |
|  | 1 | 100 |  |  |  |  |  |  |  |
|  |  |  |  |  |  |  |  |  |  |
| **Labeled Stocks:** | |  |  | Norcotinine-pyridyl-d4 |  |  |  |  |  |
|  |  |  |  |  |  |  |  |  |  |
| **Stock NCDO Prep:** | | **114.7864** | ug/mL |  |  |  |  |  |  |
|  | **Weight, mg/mL** | **% Purity** | **Corrected weight** | **Flask Vol., mL** | **Conc., mg/mL** | **Mol. Wt.Native** | **Mol. Wt. Labeled** | **Mol.wt adjust** |  |
|  | 12 | 0.98 | 11.76 | 100 | 0.1176 | 163.19 | 167.19 | 0.976075 |  |
| **Stock NCDA Prep:** | | **20** | ug/mL |  |  |  | 2 | ug/mL |  |
|  | **Vol. NCD0** | **Vol., mL** | combine all ISTD solns to a volume of 5 liter | | | | **Vol., mL** |  |  |
|  | 87.11831 | 500 |  |  |  |  | 5000 |  |  |
|  |  |  |  |  |  |  |  |  |  |
| **level** | **Native, ng / mL** | **Final Vol.** | **Native Total ng** | **Native Stock, ug/mL** | **Vol. of Native Stock, mL** | **ISTD Vol NCDA, mL** | **ISTD Vol Combined ISTD, mL** | **STD ISTD conc. ng/mL** | **spike ISTD, ng/ 50uL** |
|  |  | Use 500 mL |  | 0.02 |  | Use 50 mL |  |  |  |
| **1** | 0 | 100 | 0 |  | **0** | 5 | 50 | 1000 | 100 |
| **2** | 0.01 | 100 | 1 | NC3 | **0.05** | 5 | 50 | 1000 | 100 |
| **3** | 0.05 | 100 | 5 | NC3 | **0.25** | 5 | 50 | 1000 | 100 |
| **4** | 0.1 | 100 | 10 | NC3 | **0.5** | 5 | 50 | 1000 | 100 |
| **5** | 0.2 | 100 | 20 | NC3 | **1** | 5 | 50 | 1000 | 100 |
| **6** | 0.5 | 100 | 50 | NC3 | **2.5** | 5 | 50 | 1000 | 100 |
|  |  |  |  | 2 | 4.3 |  |  |  |  |
| **7** | 1 | 100 | 100 | NC2 | **0.05** | 5 | 50 | 1000 | 100 |
| **8** | 4 | 100 | 400 | NC2 | **0.2** | 5 | 50 | 1000 | 100 |
| **9** | 10 | 100 | 1000 | NC2 | **0.5** | 5 | 50 | 1000 | 100 |
| **10** | 20 | 100 | 2000 | NC2 | **1** | 5 | 50 | 1000 | 100 |
| **11** | 40 | 100 | 4000 | NC2 | **2** | 5 | 50 | 1000 | 100 |
| **12** | 80 | 100 | 8000 | NC2 | **4** | 5 | 50 | 1000 | 100 |
|  |  |  |  | 50 | 7.75 |  |  |  |  |
| **13** | 150 | 100 | 15000 | NC1 | **0.3** | 5 | 50 | 1000 | 100 |
| **14** | 300 | 100 | 30000 | NC1 | **0.6** | 5 | 50 | 1000 | 100 |
| **15** | 500 | 100 | 50000 | NC1 | **1** | 5 | 50 | 1000 | 100 |
| **16** | 1000 | 100 | 100000 | NC1 | **2** | 5 | 50 | 1000 | 100 |
|  |  |  |  |  | 3.9 | 80 |  |  |  |
|  | Spiking Solution Prep: |  |  |  |  |  |  |  |  |
|  | **Final Vol.(mL) of combined ISTD** | **Spike (ng)** | **Spike Vol.(uL) (mL/20)** | **ng in 5.0 L combined ISTD** | **Conc. of ISTD in combined ISTD, ng/mL** | **mL of combined ISTD needed for 100ng/ 1.0 mL of urine** |  |  |  |
|  | 5000.00 | 100 | 50 | 10000000 | 2000 | **0.05** |  |  |  |

|  |  |  | **COTININE** | | **MW= 176.1** | | |  |  |  |  |
| --- | --- | --- | --- | --- | --- | --- | --- | --- | --- | --- | --- |
|  |  |  |  |  | |  |  | |  |  |  |
| **Native Stocks:** | |  |  |  | |  |  | |  |  |  |
| **Stock CO Prep:** | | **598.486** | ug/mL |  | |  |  | |  |  |  |
|  | **Weight, mg/mL** | **% Purity** | **Corrected weight** | **Flask Vol., mL** | | **Conc., mg/mL** |  | |  |  |  |
|  | 61.07 | 0.98 | 59.8486 | 100 | | 0.598486 |  | |  |  |  |
| **Stock C1 Prep:** | | **200** | ug/mL |  | |  |  | |  |  |  |
|  | Vol CO | Vol., mL |  |  | |  |  | |  |  |  |
|  | 33.417657 | 100 |  |  | |  |  | |  |  |  |
| **Stock C2 Prep:** | | **20** | ug/mL |  | |  |  | |  |  |  |
|  | Vol C1 | Vol., mL |  |  | |  |  | |  |  |  |
|  | 10 | 100 |  |  | |  |  | |  |  |  |
| **Stock C3 Prep:** | | **0.1** | ug/mL |  | |  |  | |  |  |  |
|  | Vol C2 | Vol., mL |  |  | |  |  | |  |  |  |
|  | 0.5 | 100 |  |  | |  |  | |  |  |  |
|  |  |  |  |  | |  |  | |  |  |  |
| **Labeled Stocks:** | |  | cotinine-methyl-d3 |  | |  |  | |  |  |  |
|  |  |  |  |  | |  |  | |  |  |  |
| **Stock CDO Prep:** | | **115.6302** | ug/mL |  | |  |  | |  |  |  |
|  | **Weight, mg/mL** | **% Purity** | **Corrected weight** | **Flask Vol., mL** | | **Conc., mg/mL** | **Mol. Wt.Native** | | **Mol. Wt. Labeled** | **Mol.wt adjust** |  |
|  | 12 | 0.98 | 11.76 | 100 | | 0.1176 | 176.1 | | 179.1 | 0.98325 |  |
| **Stock CDA Prep:** | | **20** | ug/mL |  | |  |  | | 2 | ug/mL |  |
|  | **Vol. CD0** | **Vol., mL** | combine all ISTD solns to a volume of 5 liter | | | | | | **Vol., mL** |  |  |
|  | 86.482634 | 500 |  |  | |  |  | | 5000 |  |  |
|  |  |  |  |  | |  |  | |  |  |  |
| **level** | **Native, ng / mL** | **Final Vol.** | **Native Total ng** | **Native Stock, ug/mL** | | **Vol. of Native Stock, mL** | **ISTD Vol CDA, mL** | | **ISTD Vol Combined ISTD, mL** | **STD ISTD conc. ng/mL** | **spike ISTD, ng/ 50uL** |
|  |  | Use 500 mL |  | **0.1** | |  |  | |  |  |  |
| **1** | 0 | 100 | 0 |  | | **0** | 5 | | 50 | 1000 | 100 |
| **2** | 0.05 | 100 | 5 | C3 | | **0.05** | 5 | | 50 | 1000 | 100 |
| **3** | 0.2 | 100 | 20 | C3 | | **0.2** | 5 | | 50 | 1000 | 100 |
| **4** | 0.5 | 100 | 50 | C3 | | **0.5** | 5 | | 50 | 1000 | 100 |
| **5** | 1 | 100 | 100 | C3 | | **1** | 5 | | 50 | 1000 | 100 |
| **6** | 5 | 100 | 500 | C3 | | **5** | 5 | | 50 | 1000 | 100 |
|  |  |  |  | **20** | | 6.75 |  | |  |  |  |
| **7** | 20 | 100 | 2000 | C2 | | **0.1** | 5 | | 50 | 1000 | 100 |
| **8** | 40 | 100 | 4000 | C2 | | **0.2** | 5 | | 50 | 1000 | 100 |
| **9** | 80 | 100 | 8000 | C2 | | **0.4** | 5 | | 50 | 1000 | 100 |
| **10** | 200 | 100 | 20000 | C2 | | **1** | 5 | | 50 | 1000 | 100 |
| **11** | 600 | 100 | 60000 | C2 | | **3** | 5 | | 50 | 1000 | 100 |
| **12** | 1200 | 100 | 120000 | C2 | | **6** | 5 | | 50 | 1000 | 100 |
|  |  |  |  | **200** | | 10.7 |  | |  |  |  |
| **13** | 2000 | 100 | 200000 | C1 | | **1** | 5 | | 50 | 1000 | 100 |
| **14** | 4000 | 100 | 400000 | C1 | | **2** | 5 | | 50 | 1000 | 100 |
| **15** | 6000 | 100 | 600000 | C1 | | **3** | 5 | | 50 | 1000 | 100 |
| **16** | 8000 | 100 | 800000 | C1 | | **4** | 5 | | 50 | 1000 | 100 |
|  |  |  |  |  | | 10 | 80 | |  |  |  |
|  | Spiking Solution Prep: |  |  |  | |  |  | |  |  |  |
|  | **Final Vol.(mL) of combined ISTD** | **Spike (ng)** | **Spike Vol.(uL) (mL/20)** | **ng in 5.0 L combined ISTD** | | **Conc. of ISTD in combined ISTD, ng/mL** | **mL of combined ISTD needed for 100ng/ 1.0 mL of urine** | |  |  |  |
|  | 5000.00 | 100 | 50 | 10000000 | | 2000 | **0.05** | |  |  |  |

|  |  |  | **NORNICOTINE** | | **MW = 148.20** | |  |  |  |
| --- | --- | --- | --- | --- | --- | --- | --- | --- | --- |
|  |  |  |  |  |  |  |  |  |  |
| **Native Stocks:** |  |  |  |  |  |  |  |  |  |
| **Stock NN0 Prep:** | | **376.418** | ug/mL |  |  |  |  |  |  |
|  | **Weight, mg/mL** | **% Purity** | **Corrected weight** | **Flask Vol., mL** | **Conc., mg/mL** |  |  |  |  |
|  | 38.41 | 0.98 | 37.6418 | 100 | 0.376418 |  |  |  |  |
| **Stock NN1 Prep:** | | **200** | ug/mL |  |  |  |  |  |  |
|  | Vol NN0 | Vol., mL |  |  |  |  |  |  |  |
|  | 53.13242 | 100 |  |  |  |  |  |  |  |
| **Stock NN2 Prep:** | | **10** | ug/mL |  |  |  |  |  |  |
|  | Vol NN1 | Vol., mL |  |  |  |  |  |  |  |
|  | 5 | 100 |  |  |  |  |  |  |  |
| **Stock NN3 Prep:** | | **0.1** | ug/mL |  |  |  |  |  |  |
|  | Vol NN2 | Vol., mL |  |  |  |  |  |  |  |
|  | 1 | 100 |  |  |  |  |  |  |  |
|  |  |  |  |  |  |  |  |  |  |
| **Labeled Stocks:** |  |  | Nornicotine-pyridyl-d4 |  |  |  |  |  |  |
|  |  |  |  |  |  |  |  |  |  |
| **Stock NND0 Prep:** | | **75.11227** | ug/mL |  |  |  |  |  |  |
|  | **Weight, mg/mL** | **% Purity** | **Corrected weight** | **Flask Vol., mL** | **Conc., mg/mL** | **Mol. Wt.Native** | **Mol. Wt. Labeled** | **Mol.wt adjust** |  |
|  | 7.87 | 0.98 | 7.7126 | 100 | 0.077126 | 149.2 | 153.2 | 0.97389 |  |
| **Stock NNDA Prep:** | | **10** | ug/mL |  |  |  | 1 | ug/mL |  |
|  | **Vol. NND0** | **Vol., mL** | combine all ISTD solns to a volume of 5 liter | | | | **Vol., mL** |  |  |
|  | 66.56702 | 500 |  |  |  |  | 5000 |  |  |
|  |  |  |  |  |  |  |  |  |  |
| **level** | **Native, ng / mL** | **Final Vol.** | **Native Total ng** | **Native Stock, ug/mL** | **Vol. of Native Stock, mL** | **ISTD Vol NNDA, mL** | **ISTD Vol Combined ISTD, mL** | **STD ISTD conc. ng/mL** | **spike ISTD, ng/ 50uL** |
|  |  | Use 500 mL |  | 0.1 |  | Use 50 mL |  |  |  |
| **1** | 0 | 100 | 0 |  | **0** | 5 | 50 | 500 | 50 |
| **2** | 0.05 | 100 | 5 | NN3 | **0.05** | 5 | 50 | 500 | 50 |
| **3** | 0.2 | 100 | 20 | NN3 | **0.2** | 5 | 50 | 500 | 50 |
| **4** | 0.5 | 100 | 50 | NN3 | **0.5** | 5 | 50 | 500 | 50 |
| **5** | 1 | 100 | 100 | NN3 | **1** | 5 | 50 | 500 | 50 |
| **6** | 5 | 100 | 500 | NN3 | **5** | 5 | 50 | 500 | 50 |
|  |  |  |  | 10 | 6.75 |  |  |  |  |
| **7** | 10 | 100 | 1000 | NN2 | **0.1** | 5 | 50 | 500 | 50 |
| **8** | 20 | 100 | 2000 | NN2 | **0.2** | 5 | 50 | 500 | 50 |
| **9** | 50 | 100 | 5000 | NN2 | **0.5** | 5 | 50 | 500 | 50 |
| **10** | 100 | 100 | 10000 | NN2 | **1** | 5 | 50 | 500 | 50 |
| **11** | 200 | 100 | 20000 | NN2 | **2** | 5 | 50 | 500 | 50 |
| **12** | 500 | 100 | 50000 | NN2 | **5** | 5 | 50 | 500 | 50 |
|  |  |  |  | 200 | 8.8 |  |  |  |  |
| **13** | 800 | 100 | 80000 | NN1 | **0.4** | 5 | 50 | 500 | 50 |
| **14** | 1200 | 100 | 120000 | NN1 | **0.6** | 5 | 50 | 500 | 50 |
| **15** | 1600 | 100 | 160000 | NN1 | **0.8** | 5 | 50 | 500 | 50 |
| **16** | 2000 | 100 | 200000 | NN1 | **1** | 5 | 50 | 500 | 50 |
|  |  |  |  |  | 2.8 | 80 |  |  |  |
|  | Spiking Solution Prep: |  |  |  |  |  |  |  |  |
|  | **Final Vol.(mL) of combined ISTD** | **Spike (ng)** | **Spike Vol.(uL) (mL/20)** | **ng in 5.0 L combined ISTD** | **Conc. of ISTD in combined ISTD, ng/mL** | **mL of combined ISTD needed for 100ng/ 1.0 mL of urine** |  |  |  |
|  | 5000.00 | 100 | 50 | 10000000 | 2000 | **0.05** |  |  |  |

|  |  | **ANATABINE** | |  | | **MW = 160.21** | | |  |  |  |
| --- | --- | --- | --- | --- | --- | --- | --- | --- | --- | --- | --- |
|  |  |  |  |  |  | |  |  | |  |  |
| **Native Stocks:** |  |  |  |  |  | |  |  | |  |  |
| **Stock AT0 Prep:** | | **197.372** | ug/mL |  |  | |  |  | |  |  |
|  | **Weight, mg/mL** | **% Purity** | **Corrected weight** | **Flask Vol., mL** | **Conc., mg/mL** | |  |  | |  |  |
|  | 20.14 | 0.98 | 19.7372 | 100 | 0.197372 | |  |  | |  |  |
| **Stock AT1 Prep:** | | **10** | ug/mL |  |  | |  |  | |  |  |
|  | Vol AT0 | Vol., mL |  |  |  | |  |  | |  |  |
|  | 5.066575 | 100 |  |  |  | |  |  | |  |  |
| **Stock AT2 Prep:** | | **1** | ug/mL |  |  | |  |  | |  |  |
|  | Vol AT1 | Vol., mL |  |  |  | |  |  | |  |  |
|  | 10 | 100 |  |  |  | |  |  | |  |  |
| **Stock AT3 Prep:** | | **0.1** | ug/mL |  |  | |  |  | |  |  |
|  | Vol AT2 | Vol., mL |  |  |  | |  |  | |  |  |
|  | 10 | 100 |  |  |  | |  |  | |  |  |
|  |  |  |  |  |  | |  |  | |  |  |
| **Labeled Stocks:** |  |  |  |  |  | |  |  | |  |  |
|  |  |  |  |  |  | |  |  | |  |  |
| **Stock ATD0 Prep:** | | **95.6099** | ug/mL |  |  | |  |  | |  |  |
|  | **Weight, mg/mL** | **% Purity** | **Corrected weight** | **Flask Vol., mL** | **Conc., mg/mL** | | **Mol. Wt.Native** | **Mol. Wt. Labeled** | | **Mol.wt adjust** |  |
|  | 10 | 0.98 | 9.8 | 100 | 0.098 | | 161.21 | 165.24 | | 0.975611 |  |
| **Stock ATDA Prep:** | | **10** | ug/mL |  |  | |  | 1 | | ug/mL |  |
|  | **Vol. ATD0** | **Vol., mL** | combine all ISTD solns to a volume of 5 liter | | | | | **Vol., mL** | |  |  |
|  | 52.29584 | 500 |  |  |  | |  | 5000 | |  |  |
|  |  |  |  |  |  | |  |  | |  |  |
| **level** | **Native, ng / mL** | **Final Vol.** | **Native Total ng** | **Native Stock, ug/mL** | **Vol. of Native Stock, mL** | | **ISTD Vol ATDA, mL** | **ISTD Vol Combined ISTD, mL** | | **STD ISTD conc. ng/mL** | **spike ISTD, ng/ 50uL** |
|  |  | Use 500 mL |  | 0.1 |  | | Use 50 mL |  | |  |  |
| **1** | 0 | 100 | 0 |  | **0** | | 5 | 50 | | 500 | 50 |
| **2** | 0.05 | 100 | 5 | AT3 | **0.05** | | 5 | 50 | | 500 | 50 |
| **3** | 0.1 | 100 | 10 | AT3 | **0.1** | | 5 | 50 | | 500 | 50 |
| **4** | 0.2 | 100 | 20 | AT3 | **0.2** | | 5 | 50 | | 500 | 50 |
| **5** | 0.4 | 100 | 40 | AT3 | **0.4** | | 5 | 50 | | 500 | 50 |
| **6** | 0.6 | 100 | 60 | AT3 | **0.6** | | 5 | 50 | | 500 | 50 |
|  |  |  |  | 1 | 1.35 | |  |  | |  |  |
| **7** | 1 | 100 | 100 | AT2 | **0.1** | | 5 | 50 | | 500 | 50 |
| **8** | 2 | 100 | 200 | AT2 | **0.2** | | 5 | 50 | | 500 | 50 |
| **9** | 3 | 100 | 300 | AT2 | **0.3** | | 5 | 50 | | 500 | 50 |
| **10** | 4 | 100 | 400 | AT2 | **0.4** | | 5 | 50 | | 500 | 50 |
| **11** | 8 | 100 | 800 | AT2 | **0.8** | | 5 | 50 | | 500 | 50 |
| **12** | 12 | 100 | 1200 | AT2 | **1.2** | | 5 | 50 | | 500 | 50 |
|  |  |  |  | 10 | 3 | |  |  | |  |  |
| **13** | 20 | 100 | 2000 | AT1 | **0.2** | | 5 | 50 | | 500 | 50 |
| **14** | 30 | 100 | 3000 | AT1 | **0.3** | | 5 | 50 | | 500 | 50 |
| **15** | 40 | 100 | 4000 | AT1 | **0.4** | | 5 | 50 | | 500 | 50 |
| **16** | 50 | 100 | 5000 | AT1 | **0.5** | | 5 | 50 | | 500 | 50 |
|  |  |  |  |  | 1.4 | | 80 |  | |  |  |
|  | Spiking Solution Prep: |  |  |  |  | |  |  | |  |  |
|  | **Final Vol.(mL) of combined ISTD** | **Spike (ng)** | **Spike Vol.(uL) (mL/20)** | **ng in 5.0 L combined ISTD** | **Conc. of ISTD in combined ISTD, ng/mL** | | **mL of combined ISTD needed for 100ng/ 1.0 mL of urine** |  | |  |  |
|  | 5000.00 | 100 | 50 | 10000000 | 2000 | | **0.05** |  | |  |  |

|  |  | **ANABASINE** | | **MW = 162.23** | | |  |  |  |  |
| --- | --- | --- | --- | --- | --- | --- | --- | --- | --- | --- |
|  |  |  |  |  |  |  | |  |  |  |
| **Native Stocks:** |  |  |  |  |  |  | |  |  |  |
| **Stock ABO Prep:** | | **365.736** | ug/mL |  |  |  | |  |  |  |
|  | **Weight, mg/mL** | **% Purity** | **Corrected weight** | **Flask Vol., mL** | **Conc., mg/mL** |  | |  |  |  |
|  | 37.32 | 0.98 | 36.5736 | 100 | 0.365736 |  | |  |  |  |
| **Stock AB1 Prep:** | | **10** | ug/mL |  |  |  | |  |  |  |
|  | Vol ABO | Vol., mL |  |  |  |  | |  |  |  |
|  | 2.734213 | 100 |  |  |  |  | |  |  |  |
| **Stock AB2 Prep:** | | **1** | ug/mL |  |  |  | |  |  |  |
|  | Vol AB1 | Vol., mL |  |  |  |  | |  |  |  |
|  | 10 | 100 |  |  |  |  | |  |  |  |
| **Stock AB3 Prep:** | | **0.1** | ug/mL |  |  |  | |  |  |  |
|  | Vol AB2 | Vol., mL |  |  |  |  | |  |  |  |
|  | 10 | 100 |  |  |  |  | |  |  |  |
|  |  |  |  |  |  |  | |  |  |  |
| **Labeled Stocks:** |  | Anabasine-pyridyl-d4 |  |  |  |  | |  |  |  |
|  |  |  |  |  |  |  | |  |  |  |
| **Stock ABDO Prep:** | | **114.7665** | ug/mL |  |  |  | |  |  |  |
|  | **Weight, mg/mL** | **% Purity** | **Corrected weight** | **Flask Vol., mL** | **Conc., mg/mL** | **Mol. Wt.Native** | | **Mol. Wt. Labeled** | **Mol.wt adjust** |  |
|  | 12 | 0.98 | 11.76 | 100 | 0.1176 | 163.23 | | 167.26 | 0.975906 |  |
| **Stock ABDA Prep:** | | **10** | ug/mL |  |  |  | | 1 | ug/mL |  |
|  | **Vol ABDO** | **Vol., mL** | combine all ISTD solns to a volume of 5 liter | | | | | **Vol., mL** |  |  |
|  | 43.56671 | 500 |  |  |  |  | | 5000 |  |  |
|  |  |  |  |  |  |  | |  |  |  |
| **level** | **Native, ng / mL** | **Final Vol.** | **Native Total ng** | **Native Stock, ug/mL** | **Vol. of Native Stock, mL** | **ISTD Vol ABDA, mL** | | **ISTD Vol Combined ISTD, mL** | **STD ISTD conc. ng/mL** | **spike ISTD, ng/ 50uL** |
|  |  | Use 500 mL |  | 0.1 |  | Use 50 mL | |  |  |  |
| **1** | 0 | 100 | 0 |  | **0** | 5 | | 50 | 500 | 50 |
| **2** | 0.05 | 100 | 5 | AB3 | **0.05** | 5 | | 50 | 500 | 50 |
| **3** | 0.1 | 100 | 10 | AB3 | **0.1** | 5 | | 50 | 500 | 50 |
| **4** | 0.2 | 100 | 20 | AB3 | **0.2** | 5 | | 50 | 500 | 50 |
| **5** | 0.4 | 100 | 40 | AB3 | **0.4** | 5 | | 50 | 500 | 50 |
| **6** | 0.6 | 100 | 60 | AB3 | **0.6** | 5 | | 50 | 500 | 50 |
|  |  |  |  | 1 | 1.35 |  | |  |  |  |
| **7** | 1 | 100 | 100 | AB2 | **0.1** | 5 | | 50 | 500 | 50 |
| **8** | 2 | 100 | 200 | AB2 | **0.2** | 5 | | 50 | 500 | 50 |
| **9** | 3 | 100 | 300 | AB2 | **0.3** | 5 | | 50 | 500 | 50 |
| **10** | 4 | 100 | 400 | AB2 | **0.4** | 5 | | 50 | 500 | 50 |
| **11** | 8 | 100 | 800 | AB2 | **0.8** | 5 | | 50 | 500 | 50 |
| **12** | 12 | 100 | 1200 | AB2 | **1.2** | 5 | | 50 | 500 | 50 |
|  |  |  |  | 10 | 3 |  | |  |  |  |
| **13** | 20 | 100 | 2000 | AB1 | **0.2** | 5 | | 50 | 500 | 50 |
| **14** | 30 | 100 | 3000 | AB1 | **0.3** | 5 | | 50 | 500 | 50 |
| **15** | 40 | 100 | 4000 | AB1 | **0.4** | 5 | | 50 | 500 | 50 |
| **16** | 50 | 100 | 5000 | AB1 | **0.5** | 5 | | 50 | 500 | 50 |
|  |  |  |  |  | 1.4 | 80 | |  |  |  |
|  | Spiking Solution Prep: |  |  |  |  |  | |  |  |  |
|  | **Final Vol.(mL) of combined ISTD** | **Spike (ng)** | **Spike Vol.(uL) (mL/20)** | **ng in 5.0 L combined ISTD** | **Conc. of ISTD in combined ISTD, ng/mL** | **mL of combined ISTD needed for 100ng/ 1.0 mL of urine** | |  |  |  |
|  | 5000.00 | 100 | 50 | 10000000 | 2000 | **0.05** | |  |  |  |

|  |  |  | **NICOTINE** | | **MW = 162.23** | |  |  |  |
| --- | --- | --- | --- | --- | --- | --- | --- | --- | --- |
|  |  |  |  |  |  |  |  |  |  |
| **Native Stocks:** | |  |  |  |  |  |  |  |  |
| **Stock NO Prep:** | | **827.414** | ug/mL |  |  |  |  |  |  |
|  | **Weight, mg/mL** | **% Purity** | **Corrected weight** | **Flask Vol., mL** | **Conc., mg/mL** |  |  |  |  |
|  | 84.43 | 0.98 | 82.7414 | 100 | 0.827414 |  |  |  |  |
| **Stock N1 Prep:** | | **100** | ug/mL |  |  |  |  |  |  |
|  | Vol NO | Vol., mL |  |  |  |  |  |  |  |
|  | 12.08585 | 100 |  |  |  |  |  |  |  |
| **Stock N2 Prep:** | | **5** | ug/mL |  |  |  |  |  |  |
|  | Vol N1 | Vol., mL |  |  |  |  |  |  |  |
|  | 5 | 100 |  |  |  |  |  |  |  |
| **Stock N3 Prep:** | | **0.05** | ug/mL |  |  |  |  |  |  |
|  | Vol N2 | Vol., mL |  |  |  |  |  |  |  |
|  | 1 | 100 |  |  |  |  |  |  |  |
|  |  |  |  |  |  |  |  |  |  |
| **Labeled Stocks:** | |  | nicotine-methyl-d3 |  |  |  |  |  |  |
|  |  |  |  |  |  |  |  |  |  |
| **Stock NDO Prep:** | | **75.73409** | ug/mL |  |  |  |  |  |  |
|  | **Weight, mg/mL** | **% Purity** | **Corrected weight** | **Flask Vol., mL** | **Conc., mg/mL** | **Mol. Wt.Native** | **Mol. Wt. Labeled** | **Mol.wt adjust** |  |
|  | 7.87 | 0.98 | 7.7126 | 100 | 0.077126 | 163.23 | 166.23 | 0.981953 |  |
| **Stock NDA Prep:** | | **20** | ug/mL |  |  |  | 0.4 | ug/mL |  |
|  | **Vol. ND0** | **Vol., mL** | combine all ISTD solns to a volume of 5 liter | | | | **Vol., mL** |  |  |
|  | 26.40819 | 100 |  |  |  |  | 5000 |  |  |
|  |  |  |  |  |  |  |  |  |  |
| **level** | **Native, ng / mL** | **Final Vol.** | **Native Total ng** | **Native Stock, ug/mL** | **Vol. of Native Stock, mL** | **ISTD Vol NDA, mL** | **ISTD Vol Combined ISTD, mL** | **STD ISTD conc. ng/mL** | **spike ISTD, ng/ 50uL** |
|  |  | Use 500 mL |  | 0.05 |  | Use 50 mL |  |  |  |
| **1** | 0 | 100 | 0 |  | **0** | 5 | 50 | 1000 | 100 |
| **2** | 0.05 | 100 | 5 | N3 | **0.1** | 5 | 50 | 1000 | 100 |
| **3** | 0.1 | 100 | 10 | N3 | **0.2** | 5 | 50 | 1000 | 100 |
| **4** | 0.5 | 100 | 50 | N3 | **1** | 5 | 50 | 1000 | 100 |
| **5** | 1 | 100 | 100 | N3 | **2** | 5 | 50 | 1000 | 100 |
| **6** | 2 | 100 | 200 | N3 | **4** | 5 | 50 | 1000 | 100 |
|  |  |  |  | 5 | 7.3 |  |  |  |  |
| **7** | 5 | 100 | 500 | N2 | **0.1** | 5 | 50 | 1000 | 100 |
| **8** | 20 | 100 | 2000 | N2 | **0.4** | 5 | 50 | 1000 | 100 |
| **9** | 50 | 100 | 5000 | N2 | **1** | 5 | 50 | 1000 | 100 |
| **10** | 100 | 100 | 10000 | N2 | **2** | 5 | 50 | 1000 | 100 |
| **11** | 200 | 100 | 20000 | N2 | **4** | 5 | 50 | 1000 | 100 |
| **12** | 500 | 100 | 50000 | N2 | **10** | 5 | 50 | 1000 | 100 |
|  |  |  |  | 100 | 7.5 |  |  |  |  |
| **13** | 1000 | 100 | 100000 | N1 | **1** | 5 | 50 | 1000 | 100 |
| **14** | 2000 | 100 | 200000 | N1 | **2** | 5 | 50 | 1000 | 100 |
| **15** | 3000 | 100 | 300000 | N1 | **3** | 5 | 50 | 1000 | 100 |
| **16** | 4000 | 100 | 400000 | N1 | **4** | 5 | 50 | 1000 | 100 |
|  |  |  |  |  | 10 | 80 |  |  |  |
|  | Spiking Solution Prep: |  |  |  |  |  |  |  |  |
|  | **Final Vol.(mL) of combined ISTD** | **Spike (ng)** | **Spike Vol.(uL) (mL/20)** | **ng in 5.0 L combined ISTD** | **Conc. of ISTD in combined ISTD, ng/mL** | **mL of combined ISTD needed for 100ng/ 1.0 mL of urine** |  |  |  |
|  | 5000.00 | 100 | 50 | 10000000 | 2000 | **0.05** |  |  |  |
