# Supplementary material for: Validation of a LC-MS/MS Method for Quantifying Urinary Nicotine, Six Nicotine Metabolites and the Minor Tobacco Alkaloids—Anatabine and Anabasine—in Smokers' Urine
Source: PLoS One. 2014 Jul 11;9(7):e101816. doi: 10.1371/journal.pone.0101816 (PMC4094486; doi:10.1371/journal.pone.0101816)
Supplement: Data S2 — Mass transitions. (DOCX) [file pone.0101816.s002.docx]

| **Mass Transitions:** | |  | **Confirm** | **Quant** |
| --- | --- | --- | --- | --- |
|  |  | **Precursor ion** | **Product ion** | **Product ion** |
|  |  |  |  |  |
|  | **Period 1** |  |  |  |
|  | Cotinine oxide | 193 | 79 | 96 |
|  | d3 | 196 |  | 96 |
|  | Nicotine 1'oxide | 179 | 117 | 130 |
|  | d3 | 182 |  | 130 |
|  |  |  |  |  |
|  | **Period 2** |  |  |  |
|  | Hydroxycotinine | 193 | 134 | 80 |
|  | d3 | 196 |  | 80 |
|  | Norcotinine | 163 | 146 | 80 |
|  | d4 | 167 |  | 84 |
|  | Cotinine | 177 | 98 | 80 |
|  | d3 | 180 |  | 80 |
|  |  |  |  |  |
|  | **Period 3** |  |  |  |
|  | Nornicotine | 149 | 80 | 130 |
|  | d4 | 153 |  | 134 |
|  | Anatabine | 161 | 117 | 143 |
|  | d4 | 165 |  | 147 |
|  |  |  |  |  |
|  | **Period 4** |  |  |  |
|  | Anabasine | 163 | 130 | 118 |
|  | d4 | 167 |  | 134 |
|  | Nicotine | 163 | 117 | 130 |
|  | d3 | 166 |  | 130 |
