# Supplementary material for: Validation of a LC-MS/MS Method for Quantifying Urinary Nicotine, Six Nicotine Metabolites and the Minor Tobacco Alkaloids—Anatabine and Anabasine—in Smokers' Urine
Source: PLoS One. 2014 Jul 11;9(7):e101816. doi: 10.1371/journal.pone.0101816 (PMC4094486; doi:10.1371/journal.pone.0101816)
Supplement: Data S3 — De-tuning MS compound-specific parameters. (DOCX) [file pone.0101816.s003.docx]

MS parameter voltage settings (DP, EP, CE, CXP) are optimized using flow injection. A syringe pump is used to introduce a solution containing the native or labeled analyte. The optimal voltage settings are determined for each analyte and these optimal settings are used for the MS analysis, except for the cotinine and hydroxycotinine settings. The concentration of these analytes in smoker’s urine may exceed the linear limits of the detector, if voltage settings are optimal. Therefore, the voltage settings for cotinine and hydroxycotinine are de-tuned to give a lower response on the detector.

For cotinine settings, “CE” (Collision energy)was de-tuned from _35_ to _60_ and “CXP”(Collision exit potential) was de-tuned from __8__ to _25_.

For hydroxycotine settings, “CE” was de-tuned from _36_ to _50_ and “CXP” was de-tuned from __6__ to _25_.

The use of non-optimal settings allows determination of high concentrations of cotinine and hydroxycotinine without exceeding the linear limits of the detector. Multiple dilutions would be necessary if optimal settings were used. Low dilutions would be required to accurately determine levels of minor nicotine metabolites and tobacco alkaloids and high dilutions for cotinine and hydroxycotinine. Thus, off-setting the optimal voltage settings for cotinine and hydroxycotinine, allows quantification of both major and minor analytes in a single injection.

***Period One -Example Mass Spec Settings (cotinine-N-oxide; nicotine-N-oxide):***

***Cotinine N-oxide***

**Mass Table** *(note may adjust as needed to optimize)*

**Q1 Mass Q3 Mass Time**

193.1 79.2 80

193.1 96.3 80

196.1 96.3 80

Exp – 1 Duration – 4.201

Scan type – MRM Res Q1 - unit

Polarity – Positive Res Q3 - unit

Delay – 0 Pause between mass range 5.007

*Typical Gas, Temp & Voltage Settings:*

CAD – 6 DP - 63

CUR – 30 EP – 6

GS1 – 70 CE – 27

GS2 --40 CXP - 8

IS – 4000 IHE - on

TEM – 700

***Nicotine N-oxide***

**Mass Table** *(note may adjust as needed to optimize*

**Q1 Mass Q3 Mass Time**

179.1 117.3 80

179.1 130.3 80

182.1 130.3 80

Exp – 1 Duration – 4.201

Scan type – MRM Res Q1 - unit

Polarity – Positive Res Q3 - unit

Delay – 0 Pause between mass range 5.007

*Typical Gas, Temp & Voltage Settings:*

CAD – 6 DP - 47 TEM – 700

CUR – 30 EP – 5

GS1 – 70 CE – 29

GS2 --40 CXP - 10

IS – 4000 IHE – on

***Period Two-Example Mass Spec Settings (Hydroxycotinine, Norcotinine,***

***Cotinine )***

***Hydroxycotinine***

**Mass Table** *(note may adjust as needed to optimize)*

**Q1 Mass Q3 Mass Time**

193.1 134.3 80

193.1 80.1 80

196.1 80.2 80

Exp – 1 Duration – 2.505

Scan type – MRM Res Q1 - unit

Polarity – Positive Res Q3 - unit

Delay – 0 Pause between mass range 5.007

*Typical Gas, Temp & Voltage Settings:*

CAD – 6 DP - 59 TEM – 700

CUR – 30 EP – 4

GS1 – 70 CE – 36

GS2 --40 CXP - 6

IS – 5500 IHE - on

***Norcotinine***

**Mass Table** *(note may adjust as needed to optimize)*

**Q1 Mass Q3 Mass Time**

162.9 146.3 80

162.9 80.2 80

177.0 84.3 80

Exp – 1 Duration – 2.505

Scan type – MRM Res Q1 - unit

Polarity – Positive Res Q3 - unit

Delay – 0 Pause between mass range 5.007

*Typical Gas, Temp & Voltage Settings:*

CAD – 6 DP - 68 TEM – 700

CUR – 30 EP – 6

GS1 – 70 CE – 33

GS2 --40 CXP -7

IS – 5500 IHE - on

***Cotinine***

**Mass Table** *(note may adjust as needed to optimize)*

**Q1 Mass Q3 Mass Time**

177.0 98.3 80

177.0 80.2 80

180.0 80.2 80

Exp – 1 Duration – 2.505

Scan type – MRM Res Q1 - unit

Polarity – Positive Res Q3 - unit

Delay – 0 Pause between mass range 5.007

*Typical Gas, Temp & Voltage Settings:*

CAD – 6 DP - 78 TEM – 700

CUR – 30 EP – 7

GS1 – 70 CE – 35

GS2 --40 CXP - 8

IS – 5500 IHE - on

***Period Three- Example Mass Spec Settings (Nornicotine, Anatabine)***

***Nornicotine***

**Mass Table** *(note may adjust as needed to optimize)*

**Q1 Mass Q3 Mass Time**

149.1 80.1 80

149.1 130.1 80

152.9 134.2 80

Exp – 1 Duration – 2.002

Scan type – MRM Res Q1 - unit

Polarity – Positive Res Q3 - unit

Delay – 0 Pause between mass range 5.007

*Typical Gas, Temp & Voltage Settings:*

CAD – 6 DP - 50 TEM – 700

CUR – 30 EP – 5

GS1 – 70 CE – 23

GS2 --40 CXP - 8

IS – 5500 IHE – on

***Anatabine***

**Mass Table** *(note may adjust as needed to optimize)*

**Q1 Mass Q3 Mass Time**

161.2 116.9 80

161.2 143.1 80

165.2 147.1 80

Exp – 1 Duration – 2.002

Scan type – MRM Res Q1 - unit

Polarity – Positive Res Q3 - unit

Delay – 0 Pause between mass range 5.007

*Typical Gas, Temp & Voltage Settings:*

CAD – 6 DP - 45 TEM – 700

CUR – 30 EP – 8.0

GS1 – 70 CE – 40

GS2 --40 CXP - 8

IS – 5500 IHE - on

***Period Four- Example Mass Spec Settings (Anabasine, Nicotine)***

***Anabasine***

**Mass Table** *(note may adjust as needed to optimize)*

**Q1 Mass Q3 Mass Time**

163.2 118.2 80

163.2 130.3 80

167.2 134.3 80

Exp – 1 Duration – 3.975

Scan type – MRM Res Q1 - unit

Polarity – Positive Res Q3 - unit

Delay – 0 Pause between mass range 5.007

*Typical Gas, Temp & Voltage Settings:*

CAD – 6 DP - 64 TEM – 700

CUR – 30 EP – 8.8

GS1 – 70 CE – 37

GS2 --40 CXP - 8

IS – 5500 IHE - on

***Nicotine***

**Mass Table** *(note may adjust as needed to optimize)*

**Q1 Mass Q3 Mass Time**

163.1 117.3 80

163.1 130.3 80

166.1 130.3 80

Exp – 1 Duration – 3.975

Scan type – MRM Res Q1 - unit

Polarity – Positive Res Q3 - unit

Delay – 0 Pause between mass range 5.007

*Typical Gas, Temp & Voltage Settings:*

CAD – 6 DP - 55 TEM – 700

CUR – 30 EP – 7.2

GS1 – 70 CE – 28

GS2 --40 CXP - 10

IS – 5500 IHE - on
