# Supplementary material for: Validation of a LC-MS/MS Method for Quantifying Urinary Nicotine, Six Nicotine Metabolites and the Minor Tobacco Alkaloids—Anatabine and Anabasine—in Smokers' Urine
Source: PLoS One. 2014 Jul 11;9(7):e101816. doi: 10.1371/journal.pone.0101816 (PMC4094486; doi:10.1371/journal.pone.0101816)
Supplement: Data S8 — Acetone volume for precipitation 2. (DOCX) [file pone.0101816.s008.docx]

|  |  | Internal Standard Counts(Base,1 mL) | | | | |  |  |  |
| --- | --- | --- | --- | --- | --- | --- | --- | --- | --- |
|  |  |  | | | | |  |  |  |
| **Actone Added** | Cotinine oxide: | Cotinine: | Nicotine Oxide: | Nicotine: | Norcotinine: | Nornicotine: | Hydroxycotine: | Anabasine: | Anatabine: |
|  |  |  |  |  |  |  |  |  |  |
| 1 ml | 100% | 100% | 100% | 100% | 100% | 100% | 100% | 100% | 100% |
| 2 ml | 67% | 98% | 122% | 108% | 101% | 104% | 102% | 109% | 106% |
| 3 ml | 52% | 108% | 139% | 120% | 115% | 115% | 108% | 116% | 113% |
| 4 ml | 48% | 109% | 144% | 124% | 110% | 116% | 112% | 115% | 117% |
| 5 ml | 40% | 114% | 142% | 130% | 113% | 113% | 105% | 123% | 121% |

**2 mL of acetone is used for precipitation in this method.**

As the volume of acetone used for precipitation increases, the volume of urine removed by sampling 380 uL of the urine/acetone mixture is diminished. Thus before dry down, when 20 uL of methanolic HCL is added to the injector vial containing 380uL of urine/acetone mixture, a progressively greater dilution results between the urine and the methanolic HCL. This dilution is partially the cause of the increase in recovery demonstrated by increasing the acetone volume used for precipitation. The acetone dry-down has some concentrating effect; thus, there is a general increase in relative recovery with increasing acetone. Increasing the dilution of the sample may increase recovery of the spiking internal standard when an ion suppressive component is present. But, increasing the acetone may concentrate the suppressive components-causing increased ion suppression. Enhanced ion suppression by increasing the volume of acetone used for precipitation is demonstrated by cotinine oxide. Acetone appears to concentrate the ion suppressing interferences for cotinine oxide. Ion suppression is more frequently observed in the early part of a chromatograph. Cotinine oxide, being the first eluting analyte, demonstrates the adverse effects ion suppression may have on recovery.
