# Supplementary material for: Validation of a LC-MS/MS Method for Quantifying Urinary Nicotine, Six Nicotine Metabolites and the Minor Tobacco Alkaloids—Anatabine and Anabasine—in Smokers' Urine
Source: PLoS One. 2014 Jul 11;9(7):e101816. doi: 10.1371/journal.pone.0101816 (PMC4094486; doi:10.1371/journal.pone.0101816)
Supplement: Data S9 — Comparison of water, urine, and enzyme processed standards Table 5 . (DOCX) [file pone.0101816.s009.docx]

# Comparison of derived sample concentrations using urine-based standards , water-based standards or enzyme based standards.

|  | | Cotinine Oxide |  | |  | Nicotine Oxide | |  | |
| --- | --- | --- | --- | --- | --- | --- | --- | --- | --- |
| **Sample** | **water stds** | **urine stds** | **enzyme stds** |  | **Sample** | **water stds** | **urine stds** | **enzyme stds** |  |
| water blk | 0.008 | 0 | 0.008 |  | water blk | 0.006 | 0 | 0 |  |
| urine blk | 0.025 | 0 | 0.024 |  | urine blk | 0.103 | 0 | 0.063 |  |
| pool A | 1.403 | 1.028 | 1.384 |  | pool A | 0.448 | 0 | 0.244 |  |
| pool B | 11.642 | 11.221 | 11.489 |  | pool B | 20.59 | 18.645 | 20.5 |  |
| pool C | 45.148 | 44.579 | 44.561 |  | pool C | 40.893 | 39.38 | 40.917 |  |
| pool D | 209.58 | 208.285 | 206.862 |  | pool D | 396.844 | 402.905 | 398.881 |  |
| pool E | 108.647 | 107.798 | 107.237 |  | pool E | 96.486 | 96.156 | 96.825 |  |
| pool F | 403.956 | 401.803 | 398.718 |  | pool F | 401.979 | 408.15 | 404.045 |  |
| pool G | 56.307 | 55.689 | 55.576 |  | pool G | 50.663 | 49.357 | 50.742 |  |
| pool H | 117.802 | 116.912 | 116.273 |  | pool H | 398.879 | 404.983 | 400.927 |  |
| pool I | 15.934 | 15.494 | 15.726 |  | pool I | 215.372 | 217.572 | 216.383 |  |
| pool J | 66.373 | 65.71 | 65.511 |  | pool J | 104.453 | 104.293 | 104.837 |  |
| pool K | 21.701 | 21.235 | 21.418 |  | pool K | 157.721 | 158.694 | 158.407 |  |
| pool L | 182.063 | 180.889 | 179.701 |  | pool L | 122.385 | 122.606 | 122.87 |  |
| pool M | 64.142 | 63.49 | 63.309 |  | pool M | 44.158 | 42.714 | 44.201 |  |
| pool N | 3.777 | 3.39 | 3.726 |  | pool N | 0 | 0 | 0 |  |
| pool O | 555.724 | 552.901 | 548.519 |  | pool O | 918.928 | 936.098 | 923.915 |  |
| pool P | 70.222 | 69.542 | 69.31 |  | pool P | 78.122 | 77.401 | 78.357 |  |
| pool Q | 176.389 | 175.24 | 174.101 |  | pool Q | 208.315 | 210.364 | 209.286 |  |
| pool R | 409.185 | 407.009 | 403.88 |  | pool R | 580.988 | 590.968 | 584.066 |  |
|  |  |  |  |  |  |  |  |  |  |
|  |  |  |  |  |  |  |  |  |  |
|  |  | Hydroxycotinine |  |  |  |  | Norcotinine |  |  |
| **Sample** | **water stds** | **urine stds** | **enzyme stds** |  | **Sample** | **water stds** | **urine stds** | **enzyme stds** |  |
| water blk | No Peak | 0 | 0 |  | water blk | 0.015 | 0 | 0.015 |  |
| urine blk | 0.062 | 0 | 0.06 |  | urine blk | 0.035 | 0 | 0.036 |  |
| pool A | 8.17 | 7.025 | 7.922 |  | pool A | 0.265 | 0 | 0.27 |  |
| pool B | 30.755 | 28.622 | 29.821 |  | pool B | 10.861 | 10.945 | 11.178 |  |
| pool C | 174.133 | 165.727 | 168.843 |  | pool C | 42.233 | 43.551 | 43.476 |  |
| pool D | 776.641 | 741.874 | 753.047 |  | pool D | 193.393 | 200.655 | 199.097 |  |
| pool E | 2122.81 | 2029.15 | 2058.32 |  | pool E | 104.195 | 107.95 | 107.267 |  |
| pool F | 4212.55 | 4027.45 | 4084.57 |  | pool F | 386.413 | 401.265 | 397.813 |  |
| pool G | 4290.16 | 4101.67 | 4159.83 |  | pool G | 26.95 | 27.667 | 27.742 |  |
| pool H | 235.8 | 224.696 | 228.636 |  | pool H | 12.883 | 13.047 | 13.261 |  |
| pool I | 1162.67 | 1111.01 | 1127.35 |  | pool I | 180.313 | 187.061 | 185.631 |  |
| pool J | 977.969 | 934.394 | 948.258 |  | pool J | 35.173 | 36.214 | 36.209 |  |
| pool K | 7157.82 | 6843.87 | 6940.37 |  | pool K | 52.356 | 54.072 | 53.898 |  |
| pool L | 878.704 | 839.471 | 852.009 |  | pool L | 39.015 | 40.207 | 40.164 |  |
| pool M | 142.133 | 135.127 | 137.815 |  | pool M | 28.847 | 29.639 | 29.696 |  |
| pool N | 96.123 | 91.13 | 93.203 |  | pool N | 1.657 | 1.38 | 1.703 |  |
| pool O | 5178.64 | 4951.27 | 5021.31 |  | pool O | 173.332 | 179.805 | 178.444 |  |
| pool P | 415.24 | 396.285 | 402.625 |  | pool P | 26.806 | 27.518 | 27.595 |  |
| pool Q | 2465.12 | 2356.48 | 2390.23 |  | pool Q | 58.013 | 59.952 | 59.722 |  |
| pool R | 4175.91 | 3992.42 | 4049.05 |  | pool R | 160.216 | 166.174 | 164.941 |  |
|  |  |  |  |  |  |  |  |  |  |
|  |  |  |  |  |  |  |  |  |  |
|  |  | Cotinine |  |  |  |  | Nornicotine |  |  |
| **Sample** | **water stds** | **urine stds** | **enzyme stds** |  | **Sample** | **water stds** | **urine stds** | **enzyme stds** |  |
| water blk | No Peak | 0 | 0 |  | water blk | < 0 | 0 | 0.008 |  |
| urine blk | 0.035 | 0 | 0 |  | urine blk | 0.011 | 0 | 0.027 |  |
| pool A | 7.892 | 6.264 | 7.666 |  | pool A | < 0 | 0 | < 0 |  |
| pool B | 28.39 | 26.822 | 28.263 |  | pool B | 9.92 | 9.419 | 10.163 |  |
| pool C | 162.905 | 161.729 | 163.427 |  | pool C | 40.366 | 39.588 | 41.113 |  |
| pool D | 807.16 | 807.859 | 810.793 |  | pool D | 194.478 | 192.301 | 197.777 |  |
| pool E | 2019.18 | 2023.41 | 2028.66 |  | pool E | 102.165 | 100.827 | 103.936 |  |
| pool F | 3832.14 | 3841.64 | 3850.37 |  | pool F | 389.428 | 385.482 | 395.957 |  |
| pool G | 4221.48 | 4232.12 | 4241.6 |  | pool G | 44.309 | 43.495 | 45.121 |  |
| pool H | 108.56 | 107.225 | 108.82 |  | pool H | 83.817 | 82.645 | 85.284 |  |
| pool I | 1014.97 | 1016.28 | 1019.61 |  | pool I | 4.781 | 4.326 | 4.939 |  |
| pool J | 338.746 | 338.081 | 340.117 |  | pool J | 27.892 | 27.228 | 28.433 |  |
| pool K | 5931.42 | 5947.03 | 5959.79 |  | pool K | 16.037 | 15.481 | 16.382 |  |
| pool L | 707.894 | 708.303 | 711.047 |  | pool L | 11.379 | 10.864 | 11.646 |  |
| pool M | 5516.25 | 5530.65 | 5542.62 |  | pool M | 39.443 | 38.674 | 40.175 |  |
| pool N | 189.271 | 188.171 | 189.921 |  | pool N | 0.399 | 0 | 0.485 |  |
| pool O | 1501.55 | 1504.26 | 1508.53 |  | pool O | 152.904 | 151.105 | 155.515 |  |
| pool P | 458.105 | 457.787 | 460.052 |  | pool P | 58.973 | 58.026 | 60.028 |  |
| pool Q | 424.379 | 423.964 | 426.164 |  | pool Q | 26.549 | 25.897 | 27.068 |  |
| pool R | 1030.97 | 1032.32 | 1035.68 |  | pool R | 63.219 | 62.234 | 64.345 |  |
|  |  |  |  |  |  |  |  |  |  |
|  |  | Anatabine | (LOD, 0.45) |  |  |  | Anabasine | (LOD, 0.6) |  |
| **Sample** | **water stds** | **urine stds** | **enzyme stds** |  | **Sample** | **water stds** | **urine stds** | **enzyme stds** |  |
| water blk | 0 | 0 | 0 |  | water blk | 0 | 0 | 0 |  |
| urine blk | 0 | 0 | 0 |  | urine blk | 0 | 0 | 0 |  |
| pool A | 0 | 0 | 0 |  | pool A | 0 | 0 | 0.033 |  |
| pool B | 0.302 | 0.445 | 0.551 |  | pool B | 0.342 | 0.068 | 0.375 |  |
| pool C | 0.901 | 1.056 | 1.137 |  | pool C | 0.828 | 0.561 | 0.859 |  |
| pool D | 1.899 | 2.073 | 2.112 |  | pool D | 1.871 | 1.621 | 1.9 |  |
| pool E | 5.449 | 5.692 | 5.581 |  | pool E | 5.015 | 4.816 | 5.036 |  |
| pool F | 25.127 | 25.751 | 24.81 |  | pool F | 23.216 | 23.311 | 23.191 |  |
| pool G | 9.169 | 9.484 | 9.217 |  | pool G | 8.525 | 8.383 | 8.537 |  |
| pool H | 19.312 | 19.824 | 19.128 |  | pool H | 18.183 | 18.197 | 18.171 |  |
| pool I | 25.762 | 26.399 | 25.431 |  | pool I | 24.468 | 24.583 | 24.44 |  |
| pool J | 10.292 | 10.629 | 10.314 |  | pool J | 9.619 | 9.494 | 9.628 |  |
| pool K | 11.856 | 12.223 | 11.842 |  | pool K | 12.185 | 12.102 | 12.188 |  |
| pool L | 19.413 | 19.927 | 19.227 |  | pool L | 18.722 | 18.745 | 18.709 |  |
| pool M | 10.597 | 10.94 | 10.612 |  | pool M | 10.804 | 10.699 | 10.811 |  |
| pool N | 0 | 0 | 0 |  | pool N | 0 | 0 | 0 |  |
| pool O | 42.049 | 43.002 | 41.346 |  | pool O | 20.875 | 20.932 | 20.856 |  |
| pool P | 8.009 | 8.301 | 8.082 |  | pool P | 9.451 | 9.323 | 9.46 |  |
| pool Q | 3.002 | 3.197 | 3.19 |  | pool Q | 18.56 | 18.58 | 18.547 |  |
| pool R | 9.152 | 9.467 | 9.2 |  | pool R | 26.964 | 27.119 | 26.929 |  |
|  |  | Nicotine |  |  |  |  |  |  |  |
| **Sample** | **water stds** | **urine stds** | **enzyme stds** |  |  |  |  |  |  |
| water blk | 0 | 0 | 0 |  |  |  |  |  |  |
| urine blk | 0 | 0 | 0 |  |  |  |  |  |  |
| pool A | 0 | 0 | 0 |  |  |  |  |  |  |
| pool B | 17.638 | 14.482 | 17.177 |  |  |  |  |  |  |
| pool C | 37.169 | 33.951 | 36.564 |  |  |  |  |  |  |
| pool D | 362.047 | 357.794 | 359.036 |  |  |  |  |  |  |
| pool E | 1094.78 | 1088.19 | 1086.34 |  |  |  |  |  |  |
| pool F | 1733.28 | 1724.66 | 1720.11 |  |  |  |  |  |  |
| pool G | 84.484 | 81.116 | 83.529 |  |  |  |  |  |  |
| pool H | 2333.27 | 2322.73 | 2315.66 |  |  |  |  |  |  |
| pool I | 33.135 | 29.93 | 32.56 |  |  |  |  |  |  |
| pool J | 171.066 | 167.422 | 169.469 |  |  |  |  |  |  |
| pool K | 3252.79 | 3239.32 | 3228.37 |  |  |  |  |  |  |
| pool L | 1525.96 | 1518 | 1514.33 |  |  |  |  |  |  |
| pool M | 130.982 | 127.466 | 129.682 |  |  |  |  |  |  |
| pool N | 12.159 | 9.021 | 11.739 |  |  |  |  |  |  |
| pool O | 3202.18 | 3188.87 | 3178.13 |  |  |  |  |  |  |
| pool P | 101.615 | 98.191 | 100.532 |  |  |  |  |  |  |
| pool Q | 676.009 | 670.754 | 670.671 |  |  |  |  |  |  |
| pool R | 935.64 | 929.558 | 928.38 |  |  |  |  |  |  |
