# Supplementary material for: Validation of a LC-MS/MS Method for Quantifying Urinary Nicotine, Six Nicotine Metabolites and the Minor Tobacco Alkaloids—Anatabine and Anabasine—in Smokers' Urine
Source: PLoS One. 2014 Jul 11;9(7):e101816. doi: 10.1371/journal.pone.0101816 (PMC4094486; doi:10.1371/journal.pone.0101816)
Supplement: Data S10 — Dilution influence on ion suppression Table 6 . (DOCX) [file pone.0101816.s010.docx]

| Sample (vol) | Dilution factor | **CotOx** | **NicOx** | **OHCot** | **NorCot** | **Cot** | **NorNic** | **AT** | **AB** | **Nic** |
| --- | --- | --- | --- | --- | --- | --- | --- | --- | --- | --- |
| U01(200) | 5 | 2.825 | 0 | 80.504 | 4.1235 | 202.21 | 0.486 | 0.0345 | 0 | 8.5085 |
| U01(100) | 10 | 3.517 | 0 | 70.925 | 4.307 | 201.42 | 0.747 | 0.0925 | 0 | 8.1875 |
| U01(050) | 20 | 3.132 | 0 | 75.168 | 4.007 | 198.84 | 0.54 | 0.007 | 0 | 8.0415 |
| U01(020) | 50 | 3.6485 | 1.606 | 82.989 | 4.0775 | 207.64 | 0.761 | 0 | 0 | 8.725 |
|  |  |  |  |  |  |  |  |  |  |  |
| Sample (vol) | Dilution factor | **CotOx** | **NicOx** | **OHCot** | **NorCot** | **Cot** | **NorNic** | **AT** | **AB** | **Nic** |
| U08(200) | 5 | 582.77 | 1001 | 5581.8 | 185.88 | 1482.9 | 150.79 | 44.909 | 20.114 | 3334.4 |
| U08(100) | 10 | 575.71 | 991.06 | 5307.9 | 186.26 | 1539.8 | 153.94 | 41.569 | 21.289 | 3516.4 |
| U08(050) | 20 | 541.83 | 977.42 | 5472.9 | 216.41 | 1586.4 | 157.4 | 38.685 | 21.085 | 3704.5 |
| U08(020) | 50 | 562.83 | 1007.7 | 5548 | 207.18 | 1529.5 | 155.84 | 41.981 | 24.045 | 3786.3 |
|  |  |  |  |  |  |  |  |  |  |  |

Ion suppression can increase calculated values as well as decrease the values. Since the calculated values are determined by the ratio of the quantification area counts to the internal standard area counts, inhibition of the internal standard by ion suppression yields higher values and inhibition of the quantification area yields lower calculated values.

In the table above, two urine samples are analyzed at four increasing dilutions. If ion suppression is influencing the calculated values, the calculated values would be expected to vary with increasing dilution. Ion suppression is generally decreased by increasing the dilution of the urine. However, when deciding on an appropriate dilution, consider that excessive dilution can result in reduced sensitivity and higher LOD and %CV.
