# Supplementary material for: Validation of a LC-MS/MS Method for Quantifying Urinary Nicotine, Six Nicotine Metabolites and the Minor Tobacco Alkaloids—Anatabine and Anabasine—in Smokers' Urine
Source: PLoS One. 2014 Jul 11;9(7):e101816. doi: 10.1371/journal.pone.0101816 (PMC4094486; doi:10.1371/journal.pone.0101816)
Supplement: Data S11 — Ruggedness testing. (DOCX) [file pone.0101816.s011.docx]

**Ruggedness test**

Ruggedness testing was performed to access the potential of experimental variables to influence results.

A control pool was analyzed while varying:

1) pH of enzyme/ ammonium acetate buffer—varied from 5.1 to 5.5

2) Enzyme incubation at 37 degree C—time varied from 18 to 42 hours

3) Enzyme concentration during hydrolysis—enzyme volume 100 µL to 200 µL. and sample volume—200 µL or 300 µL (calculations adjusted for vol.)

The results are displayed in the table below. The variations had no substantial effect on the calculated values (Nicotine N-Oxide, anabasine and anatabine are not included in this series..

| **Vial** | **pH=5.1 Enzyme, urine/enzyme,** µL | **pH=5.5 Enzyme, urine/enzyme,** µL | **Hydrolysis Incubation Time** | **CotOx** | **OHCot** | **NorCot** | **Cot** | **NorNic** | **Nic** |
| --- | --- | --- | --- | --- | --- | --- | --- | --- | --- |
| 1 | 300/100 |  | 18 hr | 35.714 | 23.683 | 43.541 | 20.831 | 34.684 | 46.581 |
| 2 | 300/100 |  | 18 hr | 37.573 | 25.8 | 42.601 | 21.351 | 32.098 | 46.8 |
| 3 | 300/100 |  | 21 hr | 36.231 | 25.369 | 45.219 | 22.042 | 31.951 | 48.794 |
| 4 | 300/100 |  | 21 hr | 37.049 | 23.477 | 42.78 | 21.42 | 33.986 | 46.557 |
| 5 | 300/100 |  | 42 hr | 36.599 | 23.499 | 42.717 | 21.494 | 33.095 | 46.28 |
| 6 | 300/100 |  | 42 hr | 37.869 | 25.667 | 41.816 | 22.504 | 32.181 | 47.188 |
|  |  |  | **Mean** | **36.839** | **24.583** | **43.112** | **21.607** | **32.999** | **47.033** |
|  |  |  |  |  |  |  |  |  |  |
| 7 |  | 200/200 | 18 hr | 36.88 | 24.41 | 41.24 | 21.703 | 32.886 | 47.592 |
| 8 |  | 200/200 | 18 hr | 38.061 | 25.894 | 43.537 | 21.711 | 35.308 | 47.089 |
| 9 |  | 200/200 | 21 hr | 37.687 | 23.145 | 44.111 | 22.138 | 33.205 | 48.04 |
| 10 |  | 200/200 | 21 hr | 37.69 | 23.338 | 42.779 | 22.026 | 33.604 | 47.344 |
| 11 |  | 200/200 | 42 hr | 37.221 | 24.828 | 41.888 | 22.101 | 34.382 | 46.608 |
| 12 |  | 200/200 | 42 hr | 36.943 | 26.621 | 44.3 | 21.554 | 33.372 | 48.483 |
|  |  |  | **Mean** | **37.414** | **24.706** | **42.976** | **21.872** | **33.793** | **47.526** |
|  |  |  |  |  |  |  |  |  |  |
| 13 |  | 200/200 | 18 hr | 36.23 | 25.806 | 43.605 | 20.131 | 34.751 | 48.6 |
| 14 |  | 200/200 | 18 hr | 36.621 | 25.536 | 42.695 | 22.737 | 32.263 | 45.674 |
| 15 |  | 200/200 | 21 hr | 36.791 | 23.594 | 39.487 | 20.993 | 34.429 | 46.378 |
| 16 |  | 200/200 | 21 hr | 37.157 | 24.026 | 43.822 | 21.93 | 34.052 | 47.391 |
| 17 |  | 200/200 | 42 hr | 36.335 | 26.714 | 41.631 | 20.532 | 34.394 | 47.442 |
| 18 |  | 200/200 | 42 hr | 37.103 | 24.212 | 44.668 | 20.971 | 32.783 | 46.86 |
|  |  |  | **Mean** | **36.706** | **24.981** | **42.651** | **21.216** | **33.779** | **47.058** |
